# Implementing Affordable Socially Assistive Pet Robots in Care Homes Before and During the COVID-19 Pandemic: Stratified Cluster Randomized Controlled Trial and Mixed Methods Study

**DOI:** 10.2196/38864

**Published:** 2022-08-24

**Authors:** Hannah Bradwell, Katie J Edwards, Rhona Winnington, Serge Thill, Victoria Allgar, Ray B Jones

**Affiliations:** 1 Centre for Health Technology University of Plymouth Plymouth United Kingdom; 2 School of Clinical Sciences Auckland University of Technology Auckland New Zealand; 3 Donders Institute for Brain, Cognition, and Behaviour Radboud University Nijmegen Netherlands

**Keywords:** social robots, companion robots, well-being, older adults, dementia, robot pets, COVID-19

## Abstract

**Background:**

Robot pets may assist in the challenges of supporting an aging population with growing dementia prevalence. Prior work has focused on the impacts of the robot seal Paro on older adult well-being, but recent studies have suggested the good acceptability and implementation feasibility of more affordable devices (Joy for All [JfA] cats and dogs).

**Objective:**

We aimed to address the limited effectiveness research on JfA devices.

**Methods:**

We conducted an 8-month, stratified, cluster randomized controlled trial in 8 care homes in Cornwall, United Kingdom. Over 4 months, 4 care homes each received 2 JfA devices (1 cat and 1 dog; intervention group), and 4 homes received care as usual (control group). Psychometrics were collected before and after the intervention to compare the change from baseline to follow-up between the groups. In the final 4 months, all 8 care homes received devices, but only qualitative data were collected owing to COVID-19 and reduced capacity. The primary outcome was neuropsychiatric symptoms (Neuropsychiatric Inventory [NPI] Nursing Home version). Care provider burden was a secondary outcome (occupational disruptiveness NPI subscale), alongside the Challenging Behavior scale, the Holden communication scale, the Campaign to End Loneliness questionnaire, and medication use. Qualitative data were collected through care staff observation calendars and end-of-study interviews to understand use, experience, and impact. We also collected demographic data and assessed dementia severity. In total, 253 residents had robot interaction opportunities, and 83 were consented for direct data collection.

**Results:**

There was a significant difference in the total change from baseline to follow-up between the intervention and control groups for NPI (*P*<.001) and occupational disruptiveness (*P*=.03). Neuropsychiatric symptoms increased in the control group and decreased in the intervention group. No significant difference was seen for communication issues or challenging behavior. For NPI subdomains, there were significant differences from baseline to follow-up in delusions (*P*=.03), depression (*P*=.01), anxiety (*P*=.001), elation (*P*=.02), and apathy (*P*=.009), all of which decreased in the intervention group and increased slightly in the control group. The summative impact results suggested that most residents (46/54, 85%) who interacted with robots experienced a positive impact. Those who interacted had significantly higher dementia severity scores (*P=*.001). The qualitative results suggested good adoption, acceptability, and suitability for subjectively lonely individuals and lack of a novelty effect through sustained use, and demonstrated that the reasons for use were entertainment, anxiety, and agitation.

**Conclusions:**

Affordable robot pets hold potential for improving the well-being of care home residents and people with dementia, including reducing neuropsychiatric symptoms and occupational disruptiveness. This work suggests no novelty effect and contributes toward understanding robot pet suitability. Moreover, interactions were more common among residents with more moderate/severe dementia and those subjectively lonely.

**Trial Registration:**

ClinicalTrials.gov NCT04168463; https://www.clinicaltrials.gov/ct2/show/NCT04168463

## Introduction

### Background

Robot pets may offer a psychosocial method of improving well-being in older adults and people with dementia. The most well researched robot pet is Paro, the robot seal [1,2]. The use of Paro for individuals in care homes or those with dementia suggests benefits of reduced agitation and depression [3], more adaptive stress responses [4], reduced loneliness [5], reduced care provider burden [4,6], and reduced psychoactive and analgesic medication use [7]. However, Paro is expensive at approximately GBP 5000 (approximately US $6000) per robot, and this limits the number of people able to benefit from interactions [8]. The impact of this cost is evident in the limited number of real-world implementations of Paro. Additionally, robot pet alternatives to Paro have received much less research interest, creating further requirement for work such as this study.

The Joy for All (JfA) cat and dog seem to be preferred over Paro by older people in the United Kingdom, are more affordable [9-12], and are now widely used [13], although there is relatively little formal research on their benefits. A longitudinal 6-month staff diary study suggested that JfA devices had potential benefits of reduced agitation, increased communication, positive experiences, and de-escalated situations [14]. Other studies of JfA devices suggested possible positive impacts [15], including for communication, with conversations being facilitated [16,17], as well as providing companionship [18,19] and improving loneliness, mental well-being, and purpose [20]. However, there were some incidences of negative responses, such as jealousy and over-attachment [14], or dislike and rejection [16]. Much of the prior work with JfA devices has been conducted with community-dwelling older adults [16-18,20] and is mainly qualitative, with small samples [17,18].

Wexler et al [21] conducted a randomized controlled trial (RCT) with a JfA cat and dog in older adults who were hospitalized. A total of 160 older adults took part, with 80 receiving animals for the duration of their hospitalization and 80 receiving 15-minute visits from a nursing student (control group). Participants who received the JfA robot pet experienced less delirium and loneliness, and fewer falls. There was no significant effect found for cognition or depression. However, the study was conducted within a hospital rather than a care home, and each participant received a robot, which would be costly for care homes, even at the more affordable price. The study also did not measure impacts on symptoms, such as agitation and anxiety, which are commonly reported outcomes for people receiving robot pets [2,14]. It is unclear at present why the participants had been hospitalized, if any participants had dementia, or if the participants usually resided in the community or in care facilities. The duration of hospitalization is also unclear.

Marsilio et al [19] conducted the most relevant study. In their study, they provided a JfA cat to 11 care home residents for 6 weeks. They measured agitation, oxygen saturation, heart rate, and medication use at baseline and following the intervention. Qualitative weekly reflections were also maintained. They observed a decrease in agitation and an increase in oxygen saturation. However, the study had a small sample, was over a short timeframe, and had no control group. The authors provided limited details on device implementation, such as quantity, intervention dose, intervention schedule, or method of use (eg, facilitated/unfacilitated interactions or individual/group sessions).

### Aims

We aimed to address the lack of longer-term real-world research by performing a study with a large sample of care home residents and exploring the effectiveness of affordable robot pets. In particular we (1) explored if affordable robot pets led to improved well-being for an intervention group in comparison with a control group; (2) aimed to provide an indication of whether robots are robust and engaging over 8 months; and (3) identified under what circumstances and for which care home residents the robot pets were used.

## Methods

### Ethics Approval and Trial Registration

This study received ethical approval from the Health Research Authority (November 13, 2019; North East – Newcastle & North Tyneside 2 REC; Integrated Research Application System number: 268571). This study was registered on ClinicalTrials.gov (November 19, 2019; reference NCT04168463), and is reported following the CONSORT 2010 statement: extension to cluster randomized trials [22].

### Research Design

**Figure 1:**
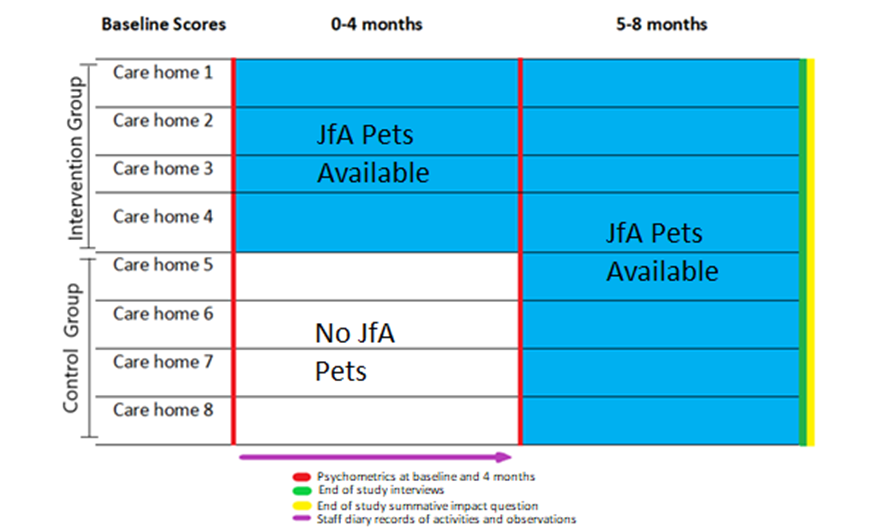
Research design and data collection. JfA: Joy for All.

This study was planned as a stepped-wedge, stratified, cluster RCT [23]. The clusters were 8 care homes. However, the trial commenced in January 2020, and the COVID-19 pandemic, resultant care home lock downs, staff workloads, and resident deaths meant that we were unable to carry out the RCT as originally planned. This variation in the planned RCT is described in Multimedia Appendix 1. The study, as conducted (Figure 1), comprised a 4-month, parallel, stratified, cluster RCT with 4 care homes in each arm. This was followed by a qualitative study over an additional 4 months, where all 8 care homes received robots, which ended with staff telephone interviews and a summative impact question. The summative impact question was a simple tool designed by the authors, where staff were asked what impact the robots had for each resident (no impact, positive impact, negative impact, or no interaction).

As demonstrated in Figure 1, the quantitative scales represented a parallel control trial, where metrics were collected for residents in the control and intervention groups at baseline and in the following 4 months. As care staff capacity was limited by the pandemic, scales were not repeated at 8 months. Diary records were maintained in both the control homes and intervention homes for the first 4 months. Due to limited staff capacity during pressures of the pandemic, diary entries were not recorded from 4 to 8 months. The qualitative impact of robots for all residents in all 8 homes was collected at 8 months through telephone interviews and a summative impact question.

### Collaborating Sites

Eight residential care facilities present in rural towns in Cornwall, comprising 4 care homes with nursing care and 4 residential-only care homes, with a total resident population of 253, agreed to collaborate before the start of the project. Sixteen care staff became collaborators for the purpose of completing scales and recording observations of residents. Homes were eligible for participation if they provided residential care or nursing to older adults, were situated in Cornwall, United Kingdom, and allowed regular researcher visits.

### Recruitment of Residents for the Collection of Individual Data

In November 2019, researchers and care staff talked to residents or residents’ relatives to gauge interest in participation. Prior to randomization, written informed consent was obtained directly from 30 individuals with the capacity to consent and from 53 authorized third parties for individuals without capacity. Where consent involved advice from a consultee of a participant, care home collaborators were encouraged to use measures of assent throughout the trial to ensure participant comfort. Care staff were asked to be mindful not to cause residents distress if they did not like the robots. The 83 care home residents recruited for directly collected data comprised 61 females and 22 males, and represented 32.8% (83/253) of all residents who had access to the robot pets. To allow stratified randomization, staff assessed consenting residents using the Dementia Severity Rating Scale [24]. This provides a score ranging from 0 to 54, with 0-18 indicating mild dementia, 19-36 indicating moderate dementia, and 37-54 indicating severe dementia.

### Randomization

The 8 care homes were stratified into 4 pairs based on the number of consented residents, average age, and average dementia severity (as key factors likely to influence behavior) using randomly permuted blocks of size 2 by HB. Each member of the pair was then randomly allocated to either group A or group B, and finally group A and group B were randomly allocated by a separate researcher (KJE) using a random number generator to the intervention or control arm in a 1:1 ratio (homes 1-4 and homes 5-8).

### Data Collection

#### Individual Participant Data

We aimed to collect pre/post data on 5 scales for 83 residents who had consented. The primary outcome was neuropsychiatric symptoms, measured with the Neuropsychiatric Inventory (NPI) by staff [25], with higher scores indicating higher symptom prevalence. Secondary outcomes were measured with the Challenging Behavior Scale [26], Holden Communication Scale [27], and NPI occupational disruptiveness subdomain scale by staff. Residents were assisted in directly completing the 3-item Campaign to End Loneliness [28] questionnaire. The 5 scales were completed at baseline (December 2019) and at 4 months (May 2020). Finally, staff indicated, through a summative impact question, whether (1) each resident had no interaction with robots, (2) robots had a negative impact, (3) robots had no impact, or (4) robots had a positive impact for all participants at 8 months, as part of an *end of study reflection*, when the intervention group had been using robots for 8 months and the control group had been using robots for 4 months.

#### Data Collection Tools for Individual Outcomes at Baseline and 4 Months

The primary outcome was assessed with the NPI Nursing Home version [25] (total score 0-120), with higher scores indicating higher symptom prevalence.

The secondary outcomes were assessed with the following: (1) Challenging Behavior Scale [26] (scored 0-400), with higher scores indicating more challenging behavior; (2) Holden Communication Scale [27] (scored 0-48), with higher scores indicating greater communication challenges; (3) Campaign to End Loneliness Measurement Tool (3-item) [28] (scored 0-12), with higher scores indicating greater loneliness; and (4) NPI subdomain scales [25] (scored 0-12) and NPI occupational disruptiveness scale (scored 0-50), with higher scores indicating more disruptiveness.

#### Cluster (Care Home) Level Data at 8 Months

**Figure 2:**
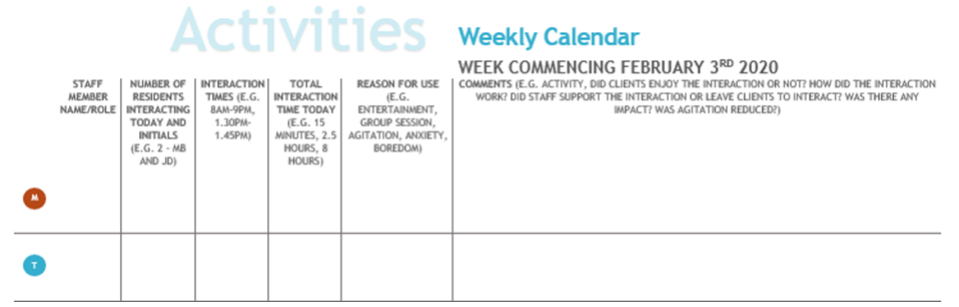
Example calendar for recording activities. Monday and Tuesday rows are shown (full page includes all days of the week).

Moyle et al [29] noted that behavioral and psychological improvements were not always shown through the chosen scales, and that an evaluation should look beyond these for a picture of overall effectiveness, including comments and observations of care staff and family members. Collaborating care staff in all homes were encouraged to record observations on their calendars using an experience sampling method [30]. Based on our previous use of diaries [14], we created wall-hung calendars for data entry (Figure 2).

Staff were asked to record notes on the calendar each time they observed resident-robot interactions, where possible. We also conducted qualitative semistructured interviews with care staff at 8 months, with open questions aiming to understand the robots’ use, engagement, and impact, and the experiences of the staff and residents (Textbox 1).

**Semistructured interview guide.**

**Questions** (text in brackets was not spoken but provided as notes for the researchers; additionally, questions on benefits were only asked if benefits were mentioned)

- Tell me about your experience with the robot pets here at (name of home)?
- How were the robot pets used?
- Was there any impact? (positive or negative impact – follow up questions included: please explain how many residents benefitted [if benefits were mentioned] and how?)
- (If benefits were mentioned above) Which residents benefited? Would you say there were residents the pets were more or less suited to based on your first-hand experience?
- Were there any particular features of the pets you perceived positively based on their use here with residents?
- Were there any particular features of the pets you perceived negatively based on their use here with residents?
- How did the residents engage with the robot pets?
- Has there been any change in their use over time?
- Has there been any change in reactions over time?
- Any additional comments or observations?
- Were there any practical considerations? (eg, robustness, cleaning, and batteries)
- How did the COVID-19 pandemic and lockdown affect use?

### Intervention

In mid-January 2020, homes in the intervention group were gifted a JfA cat and dog to keep indefinitely and to use or not use as they felt appropriate. The researcher provided infection control information [31], providing care homes with the cleaning protocol and informing them of products to use. This study aimed to respond to limitations of trials with highly controlled intervention doses, and explore robot pet effectiveness rather than efficacy [32]. The researcher discussed past research with care home staff, providing examples and ideas, including prior work that implemented robots with structured daily group or individual sessions [3,29], or used robots *when required* for reducing loneliness, anxiety, depression, or agitation, as in previous research with Paro [33]. Decisions on robot use were then left to the professional judgement of care staff. In the fourth month of the trial, the pandemic resulted in changes to the use of robots, with homes tending to reserve robots for specific individuals during specific times, rather than conducting group activities with robots passed between residents.

### Sample Size

The sample size was primarily informed by feasibility and the number of residents in each home providing consent, but we calculated the minimum number required for the total sample. Based on previous work reporting on the minimal clinically important difference for the original NPI [34], we calculated using the lower value of 2.77, with an estimated SD of 3.31. To detect a difference of 2.77 between groups, based on 80% power and 5% significance, a sample size of 25 per condition would be required, inflated by 20% to account for any loss to follow-up, resulting in a total sample size of at least 70 individuals.

### Data Analysis

Descriptive statistics are presented as mean (SD), median (IQR), and n (%). The changes from baseline to 4 months on the primary and 3 secondary ordinal scales were compared between the control and intervention groups using the Mann Whitney *U* test. SPSS 25 (IBM Corp) was used for statistical analysis. A *P* value <0.05 was considered to indicate statistical significance.

Qualitative diaries and interviews were individually subject to content analysis, and have been reported together due to great similarity of themes. Content analysis follows similar processes to thematic analysis, involving coding and categorizing of textual information; however, the frequency of occurrence is of additional importance [35].

### Quantitative Scales

For the quantitative measures, we first report the primary (NPI) and secondary psychometric outcomes (communication, challenging behavior, and occupational disruptiveness) and report intention-to-treat (ITT) results for all residents (as randomized) who survived to the 4-month follow-up (n=63). We then report NPI subdomain results, followed by the summative impact question completed by a staff member at 8 months, to indicate the overall robot impact for each consented resident (n=83). We subsequently report a comparison of the characteristics of residents who did and did not interact with robots during the study, to comment on the suitability of the devices, based on residents who survived till follow-up (n=63). This was because of the possibility that residents who died never had the opportunity to interact with the robots, rather than, for example, rejected the robots owing to a lack of suitability.

## Results

### Participants

**Figure 3:**
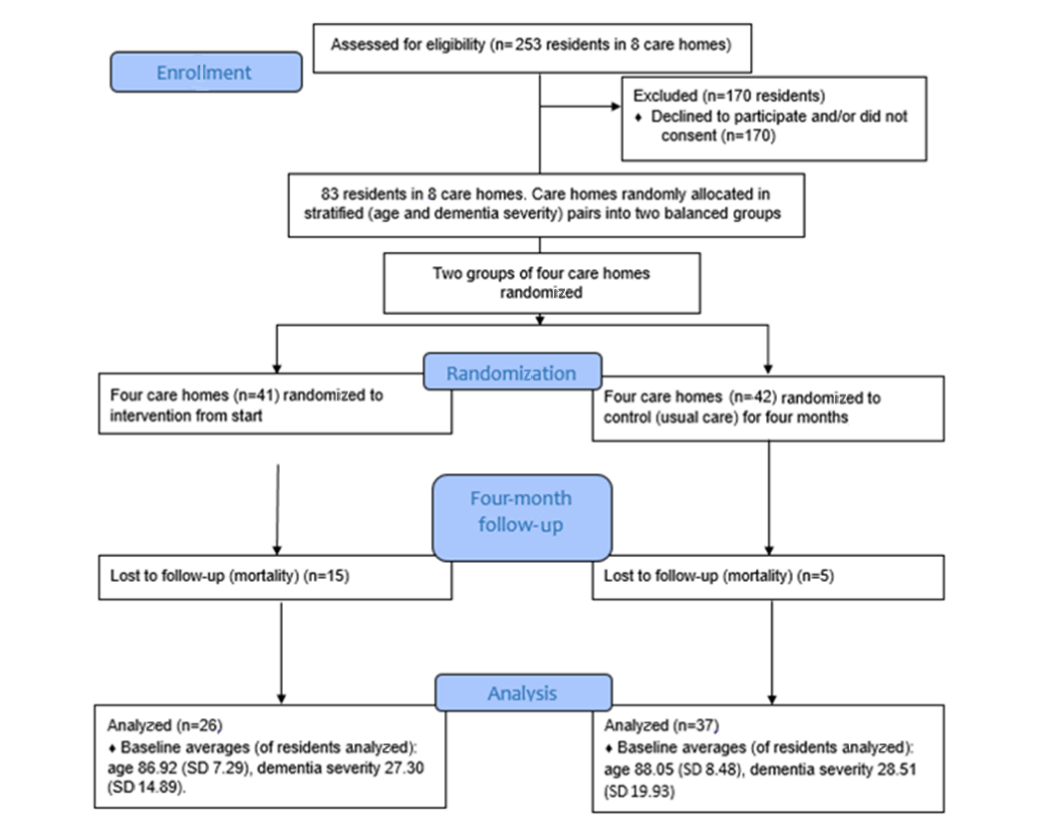
CONSORT diagram of trial recruitment, allocation, and analysis of data.

The average age of consented participants was 87.21 years (SD 7.42 years), and the average dementia score was 32.11 (SD 10.52) (Tables 1 and 2). Twenty of the 83 residents recruited died during the study, leaving 63 participants for analysis (49 females and 14 males) (Table 1; Figure 3). There was no difference in the dementia severity (U=513; n=63; *P=*.65) or age (U=549; n=63; *P=*.34) of residents included in the analysis between the intervention and control groups.

Figure 3 shows that a greater number of deaths occurred in the intervention group than in the control group. Considering our concerns on infection control and the timing of the trial in the early stages of the COVID-19 pandemic, we carried out more detailed analysis of deaths and enquired with care home staff. Further details are given in Multimedia Appendix 2.

**Table 1:** Demographic characteristics of the participating homes and consented participants.

Home	Site type	Staff collaborators (N=16)	Total residents (N=253)	Consented residents (N=83)	Gender (22 M, 61 F)^a^	Residents included in the analysis (N=63)
1^b^	Nursing	2	33	9	3 M, 6 F	3
2^b^	Residential	2	16	11	1 M, 10 F	10
3^b^	Nursing	2	36	9	4 M, 5 F	4
4^b^	Residential	2	36	12	4 M, 8 F	9
5	Nursing	2	36	7	4 M, 3 F	4
6	Residential	2	27	13	4 M, 9 F	12
7	Nursing	2	31	13	1 M, 12 F	12
8	Residential	2	38	9	1 M, 8 F	9

^a^M: male; F: female.

^b^Homes included in the intervention group (see Figure 1).

**Table 2:** Demographic characteristics of the consented participants.

Home	Age of the consented residents (years), mean (SD)	Age of the residents analyzed (years), mean (SD)	Dementia severity score for the consented residents (scored 0-54), mean (SD)	Dementia severity score for the analyzed residents (scored 0-54), mean (SD)
1^a^	87.67 (6.73)	86.33 (7.37)	40.56 (9.38)	43.33 (9.71)
2^a^	90.73 (7.85)	90.10 (7.97)	19.63 (12.82)	17.30 (10.76)
3^a^	82.89 (2.51)	83.00 (7.39)	44.11 (8.25)	37.50 (7.59)
4^a^	85.08 (6.33)	85.33 (6.1)	32.58 (15.77)	28.56 (15.58)
5	86.29 (10.05)	87.75 (9.60)	36.14 (10.07)	35.75 (7.58)
6	90.46 (9.53)	89.42 (9.14)	5.23 (5.93)	4.75 (5.93)
7	85.15 (8.34)	85.75 (8.41)	46.77 (6.13)	47.33 (6.03)
8	89.44 (8.00)	89.44 (8.00)	31.89 (15.84)	31.89 (15.84)
Overall	87.21 (7.42)	87.14 (8.00)	32.11 (10.52)	30.80 (9.88)

^a^Homes included in the intervention group (see Figure 1).

### Psychometric Analysis

The data for communication issues, challenging behavior, neuropsychiatric symptoms, and occupational disruptiveness are presented in Tables 3 and 4.

Table 4 demonstrates that based on ITT analysis, there was a significant difference in the total change in NPI and occupational disruptiveness scores between the intervention and control groups. Neuropsychiatric symptoms increased in the control group and decreased in the intervention group. No significant difference was present for communication issues or challenging behavior from baseline to follow-up between the control and intervention groups.

NPI data for the intervention and control groups are presented in Tables 5 and 6.

When looking at the individual domains, there was a significant difference between the control and intervention groups in the total change from baseline to follow-up for delusions, depression, anxiety, elation, and apathy, all of which decreased in the intervention group and increased slightly in the control group. There was no significant difference from baseline to follow-up between the 2 groups for other subdomains. Multimedia Appendix 3 presents issues in the normality of the data, justifying the choice of nonparametric analysis.

**Table 3:** Baseline and 4-month scores for communication issues, challenging behavior, neuropsychiatric symptoms, and occupational disruptiveness in the control and intervention groups.

Scale (scoring)	Intention-to-treat analysis (as randomized) (N=63)^a^										
	Baseline						Follow-up				
	Control (n=37)			Intervention (n=26)			Control (n=37)			Intervention (n=26)	
	Mean (SD)	Median (IQR)	Mean (SD)		Median (IQR)	Mean (SD)		Median (IQR)	Mean (SD)		Median (IQR)
Communication (0-48)	20.57 (15.13)	21.00 (29.5)	16.58 (11.85)		15.00 (20.75)	21.97 (15.12)		22.00 (30.00)	17.23 (15.33)		14.00 (29.75)
Challenging behavior (0-400)	54.86 (56.95)	32.00 (82.00)	43.38 (43.02)		26.00 (53.00)	48.22 (53.98)		29.00 (73.00)	31.85 (38.39)		19.50 (36.00)
Neuropsychiatric Inventory (0-120)	16.64 (16.41)	16.00 (13.50)	19.19 (17.08)		15.00 (22.50)	19.41 (18.72)		11.00 (26.00)	9.62 (7.83)		9.00 (10.75)
Neuropsychiatric Inventory occupational disruptiveness (0-50)	5.51 (6.37)	4.00 (8.00)	4.42 (4.86)		3.00 (7.00)	5.46 (6.26)		3.00 (8.50)	3.19 (4.54)		1.00 (3.25)

^a^The intention-to-treat analysis excludes the 20 residents who died but includes the 63 who potentially had access to the robots.

**Table 4:** Difference in communication issues, challenging behavior, neuropsychiatric symptoms, and occupational disruptiveness from baseline to follow-up between the control and intervention groups.

Scale (scoring)^a^	Intention-to-treat analysis (as randomized) (N=63)^b^		
	Difference (baseline to follow-up), mean (SD)		Test of difference (control vs intervention)
	Control (n=26)	Intervention (n=20)	Mann-Whitney *U* test *P* value
Communication (0-48)	1.41 (6.00)	0.65 (7.54)	.18
Challenging behavior (0-400)	−6.65 (25.65)	−11.54 (23.92)	.35
Neuropsychiatric Inventory (0-120)	2.76 (9.43)	−9.58 (14.06)	<.001
Neuropsychiatric Inventory occupational disruptiveness (0-50)	−0.05 (2.47)	−1.23 (2.53)	.03

^a^For all scales, higher scores indicate greater prevalence of challenges.

^b^The intention-to-treat analysis excludes the 20 residents who died but includes the 63 who potentially had access to the robots.

**Table 5:** Baseline and 4-month Neuropsychiatric Inventory domain data for the intervention and control groups.

Scale (scored 0-12)	Baseline					Follow-up			
	Control (n=37)		Intervention (n=26)		Control (n=37)			Intervention (n=26)	
	Mean (SD)	Median (IQR)	Mean (SD)	Median (IQR)	Mean (SD)		Median (IQR)	Mean (SD)	Median (IQR)
Delusions	0.76 (2.46)	0.00 (0.00)	1.57 (3.34)	0.00 (0.25)	1.43 (3.18)		0.00 (.50)	0.19 (0.80)	0.00 (0.00)
Hallucinations	0.49 (2.04)	0.00 (0.00)	0.73 (1.95)	0.00 (0.00)	1.03 (2.69)		0.00 (0.00)	0.27 (0.87)	0.00 (0.00)
Agitation	4.68 (3.86)	4.00 (7.50)	3.42 (4.20)	2.50 (6.00)	3.70 (4.27)		2.00 (7.00)	1.00 (2.40)	0.00 (0.25)
Depression	2.43 (3.21)	2.00 (3.00)	2.08 (2.53)	0.50 (4.50)	3.03 (2.94)		2.00 (5.00)	1.62 (3.03)	0.00 (2.50)
Anxiety	2.30 (3.19)	1.00 (3.50)	3.31 (4.25)	0.00 (8.00)	2.92 (3.55)		2.00 (6.00)	0.84 (2.12)	0.00 (0.00)
Elation	2.30 (3.19)	0.00 (0.00)	1.31 (2.65)	0.00 (2.00)	0.84 (2.28)		0.00 (0.00)	0.92 (2.61)	0.00 (0.00)
Apathy	2.24 (2.56)	2.00 (4.00)	3.58 (3.30)	4.00 (6.00)	2.76 (3.55)		2.00 (4.00)	2.38 (3.45)	0.00 (4.00)
Disinhibition	0.78 (2.76)	0.00 (0.00)	0.37 (1.30)	0.00 (0.00)	0.78 (2.76)		0.00 (0.00)	0.00 (0.00)	0.00 (0.00)
Irritability	2.62 (3.36)	1.00 (4.00)	1.54 (3.05)	0.00 (2.00)	2.59 (3.48)		0.00 (6.00)	1.19 (2.83)	0.00 (1.25)
Motor behaviors	0.14 (0.67)	0.00 (0.00)	1.31 (2.69)	0.00 (0.75)	0.32 (1.11)		0.00 (0.00)	1.19 (2.68)	0.00 (0.00)
Sleep behaviors	1.22 (2.85)	0.00 (0.50)	1.38 (2.74)	0.00 (2.25)	0.24 (1.04)		0.00 (0.00)	1.27 (2.91)	0.00 (0.50)
Eating behaviors	0.46 (1.10)	0.00 (0.00)	1.81 (4.10)	0.00 (0.00)	0.35 (0.92)		0.00 (0.00)	0.88 (2.80)	0.00 (0.00)

**Table 6:** Difference in the Neuropsychiatric Inventory domains from baseline to follow-up between the intervention and control groups.

Scale (scored 0-12)	Intention-to-treat analysis (as randomized) (N=63)			
	Difference (baseline to follow-up), mean (SD)			Test of difference (control vs intervention)
	Control (n=37)	Intervention (n=26)	Mann-Whitney *U* test *P* value	
Delusions	0.68 (2.85)	−1.38 (3.46)	.03	
Hallucinations	0.54 (1.48)	−0.46 (2.21)	.06	
Agitation	−0.97 (2.93)	−2.42 (3.76)	.22	
Depression	0.56 (2.30)	−0.46 (3.19)	.01	
Anxiety	0.62 (1.93)	−2.46 (4.37)	.001	
Elation	0.62 (2.00)	−0.38 (2.47)	.02	
Apathy	0.51 (2.43)	−1.19 (3.14)	.009	
Disinhibition	0.00 (0.00)	−0.35 (1.29)	.08	
Irritability	−0.03 (3.47)	−0.35 (3.39)	.55	
Motor behaviors	0.19 (0.81)	−0.12 (0.59)	.10	
Sleep behaviors	−0.97 (2.98)	−0.12 (0.99)	.19	
Eating behaviors	−0.11 (0.66)	−0.92 (3.26)	.34	

### Summative Impact Question

The summative question asked about care staff members’ perceptions of robot use and the impact for all residents at the 8-month time point after all homes had received robots and had been implementing them for either 4 or 8 months. Among the residents reported to interact with the robots (54/81), 85% (46/54) were reported to have positive experiences. Table 7 demonstrates that most residents who survived the 8 months and were included in the analysis (61/81) interacted with the pets (46/61, 75%), and that most (40/61, 66%) experienced a positive impact, with only 1 resident (male) experiencing a negative impact. This summative question provided the perception of 1 member of the staff in each home, and thus, there may be inaccuracies based on different staff members observing robot use with different residents, although the collaborating staff member was always the staff member in each home with the most insight and experience. Additionally, this observation may suffer from memory strain, with staff asked to reflect over the prior 8 months. However, as nearly a quarter (15/61) of the residents included in the analysis did not interact with the robots (Table 7), we performed a comparison of the characteristics of residents who did and did not interact with the robots to comment on suitability.

**Table 7:** Care staff summative estimation of the impact of robot pets for each resident at 8 months (N=83).

Care home		Total number of residents	Consented participants	Died by the 4-month follow-up	No interaction	Negative impact	No impact	Positive impact
**Intervention care home, n**								
	1	33	9	6	4	0	1	4
	2	16	11	1	2	0	0	9
	3	36	9	5	5	0	1	3
	4	36	12	3	2	0	1	9
	Overall	121	41	15	13	0	3	25
**Control care home, n**								
	5	36	7	3	0	0	1	3
	6^a^	27	13^a^	1	7	0	0	3
	7	31	13	1	0	0	3	9
	8	38	9	0	2	1	0	6
	Overall	132	42	5	9	1	4	21
All participants, n (%)		253 (100)	83 (32.8)	20 (24.1)	22 (27.2)^a^	1 (1.2)^a^	7 (8.6)^a^	46 (56.8)^a^
Residents included in the RCT^b^ analysis at 4 months (n=61^a^), n (%)		N/A^c^	N/A	N/A	15 (24.6)^a^	1 (1.6)^a^	5 (8.2)^a^	40 (65.6)^a^

^a^Data on interaction are missing for 2 people in home 6.

^b^RCT: randomized controlled trial.

^c^N/A: not applicable.

### Difference Between Interacting and Noninteracting Residents

Residents who subsequently went on to interact with robots had significantly higher dementia severity scores than residents who did not interact (Table 8). On average, residents who did interact were considered to be at the higher end of moderate dementia (19-36), while residents who did not interact were considered to have mild dementia (0-18). The interacting residents also had significantly poorer communication scores and scored significantly higher for challenging behaviors and NPI occupational disruptiveness. There was no difference in the overall NPI score, age, or gender.

**Table 8:** Baseline characteristics of residents who did and those who did not interact with robots.

Scale	Residents who did interact (n=46), mean (SD)	Residents who did not interact (n=15), mean (SD)	Mann-Whitney *U* test *P* value
Communication	22.22 (13.29)	11.20 (11.98)	.005
Challenging behavior	61.02 (54.73)	22.20 (26.27)	.003
Neuropsychiatric Inventory	20.28 (18.09)	11.40 (9.06)	.06
Neuropsychiatric Inventory occupational disruptiveness	6.15 (6.23)	2.27 (2.84)	.01
Dementia severity	33.46 (15.60)	14.73 (16.03)	.001
Age (years)	87.02 (7.68)	88.47 (9.08)	.32

The above findings suggest that robots are perhaps more suited to residents scoring higher for dementia severity, who also experience more communication issues and challenging behavior as associated symptoms.

The fact that many care homes restricted shared robot use from 4 months onwards would have influenced some residents not interacting, particularly in control homes where robots were only provided from month 5. However, homes reported aiming to allow interested participants opportunities to interact (individually after robot cleaning rather than group sessions), and robots tended to become *adopted* by residents who found particular benefit. Staff reported not pursuing interactions with residents who were disinterested, feeling they were best placed with *adoptees* in any case.

### Qualitative Calendar Entries

During the first 4 months, staff in the 4 control homes provided 139 days of calendar entries describing usual resident activities and moods. Staff in the 4 intervention homes provided 109 days of calendar entries. In total, about 25% (248/960 [8×120]) of care-home-days were captured. The diaries reported a total of 516.3 hours of interaction with the robots over the 4 months, with an average interaction length of 3.9 hours. The range of interaction lengths varied from 0.25 hours to 24 hours, where residents kept the robot with them all day and night. On average, 4 residents interacted with the robots on each reported day (range 1-8). The main reasons recorded in the *reason for use* of robots were entertainment, anxiety, and agitation (Table 9). In control homes, typical activities included singing, manicures, reminiscence, television, garden games, hairdresser visits, and quizzes.

Table 10 demonstrates the themes resulting from analysis of comments made in the calendars and interviews. The full table of themes with quotes as example evidence is available in Multimedia Appendix 4, further to a full narrative on the themes.

**Table 9:** Reported reasons for using robots (N=109).

Reason	Value, n
Entertainment	40
Anxiety	33
Agitation	31
Boredom	30
Group session	10
Company	7
Love	6
Cuddles	4
Nurturing	3
Loneliness	3
Affection	2
Stress	1
Distress	1
Distraction	1
Observation	1
Sadness	1
Reassurance	1

**Table 10:** Content analysis of qualitative interviews and calendar entries.

Theme (e*xplanation*)	Codes (interviews: n; diaries: n)
Adoption (*Evidence strongly supported good robot adoption into services, and usually by particular “adoptee” residents*)	Love (interviews: 11; diaries: 13) Ownership (interviews: 18; diaries: 6) Individual use (interviews: 9; diaries: 14) High level of usage (diaries: 12) Jealousies or possessiveness (interviews: 6; diaries: 6) No novelty (interviews: 9) Naming (interviews: 7) Group sessions (diaries: 5) Personalizing (interviews: 1)
Well-being effects, particularly mood (*Evidence strongly supported well-being benefits*)	Calming (interviews: 10; diaries: 20) Enjoyment (interviews: 1; diaries: 19) Anxiety reduced (interviews: 3; diaries: 13) Companionship (interviews: 7; diaries: 6) Smiles, happiness (interviews: 1; diaries: 9) Engaging resident (interviews: 10) Relaxing or settling (diaries: 7) Mood improved (interviews: 7) Provides a focus (interviews: 5) Distraction (interviews: 3; diaries: 2) Agitation reduced (diaries: 5) Entertainment and laughter (interviews: 1; diaries: 3) Therapeutic (interviews: 3) Reassurance (interviews: 3) Sundowner (interviews: 2) Reduced boredom (interviews: 1; diaries: 1) Enabled eating (diaries: 1)
Effects on communication (*Evidence supported robot impact on residents’ communication, with the pets and people, further to improving speech capabilities*)	Communication-pet (diaries: 25) Communication with others, and speech (interviews: 19; diaries: 2) Reminiscence (interviews: 5; diaries: 1) Interaction (interviews: 4)
Isolation and COVID (*Evidence showed particular benefits of robot pets as a supporting strategy against loneliness and isolation in response to the COVID-19 pandemic*)	COVID use (interviews: 15) Cleanliness and infection control (interviews: 9) Isolation (interviews: 5)
Design (*Staff suggested a few possible design improvements based on their experience, and commented on positive and negative design factors*)	Improvements (interviews: 11) Realistic (interviews: 9) Sound off (interviews: 8) Expectations (interviews: 8) Weight and size (interviews: 7) Breakage (interviews: 7) Battery life (interviews: 4) Importance of movement (interviews: 4) Purring as relaxing (interviews: 2; diaries: 2) Heartbeat enjoyable (interviews: 1; diaries: 2)
Suitability (*The data gave some insight into the most suitable use context for use with residents, including those with dementia and those isolated, perhaps due to mobility impairments*)	Dementia severity (interviews: 31) Limited interest (diaries: 17) Think it is real (interviews: 14) Dislike (interviews: 2; diaries: 9) Wide appeal (interviews: 7) Reduced mobility (interviews: 5; diaries: 1) Previous pets (interviews: 3; diaries: 1) Infantilizing (interviews: 4) Staff dislike (interviews: 1)
Nurture (*Evidence suggested residents tended to care for robots and treat them as living animals*)	Cuddled and fussed (diaries: 29) Feeding (interviews: 8; diaries: 5) Care for and nurture the pet (interviews: 8; diaries: 5)

## Discussion

### Overview

Our results suggest that affordable robot pets are able to produce important well-being impacts for older adult care home residents, with further potential positive impacts for staff through reduced occupational disruptiveness.

### Principal Findings and Comparison With Prior Work

This study strongly supports the usefulness and benefits of implementing affordable robot pets in care homes for older adults. It contributes toward limited literature in this area, with most prior companion robot research focusing on Paro [1,29], a device with limited acceptability among older people [9,10] and too expensive for widespread implementation [12,36]. Previous work considering alternative more affordable robots had been mainly conducted within the community [16,20] or in hospital settings [37,38], with limited generalizability to care home residents [39], and with smaller samples and short time frames [19,40]. Additionally, much previous work has involved highly controlled intervention doses [7,29], thus assessing efficacy rather than potential real-world effectiveness [32]. This study therefore provides an important and novel contribution to companion robot literature.

JfA robots demonstrated significant improvements from baseline to follow-up for the primary outcome of neuropsychiatric symptoms and secondary outcome of occupational disruptiveness based on ITT analysis between the control and intervention groups. The reduction in neuropsychiatric symptoms in the intervention group was an encouraging result, suggesting important effects of affordable robot use, as the NPI measures key behavioral and psychological symptoms associated with dementia [25]. There were no significant differences for the secondary outcomes of communication impairments and challenging behavior. The NPI subscale of occupational disruptiveness was used as an indicator of care provider burden, and the reduction seen here is congruent with results from the study by Saito et al [6] who suggested Paro could decrease care provider burden. We did not use a specific care provider burden scale, with the stigmatizing wording found to discourage carer responses in a pilot study. However, the significant difference in occupational disruptiveness could suggest that the implementation of pets aided in easing the challenges of the carer’s role.

When analyzing the individual NPI subdomains, the results suggested significant differences in the mean change from baseline to follow-up between the intervention and control groups for delusions, depression, elation, anxiety, and apathy. This finding suggests that JfA devices can achieve similar well-being outcomes to those reported for Paro, particularly around reducing depression [3,5-7]. The support for impact on delusions is also congruent with the work of Schulman-Marcus et al [38], who reported on stakeholders feeling that JfA devices were useful for hospital patients with delirium. The potential for these more affordable devices to produce promising therapeutic benefits is an important result, with implications for research and practice. Interestingly, we did not find a significant impact for agitation, as previous work did for Paro [3]. Similarly, in the cluster RCT conducted by Moyle et al [29], there was no significant effect on agitation in the Paro intervention group. Moyle et al [29] suggested that chosen psychometrics can sometimes miss behavioral improvements, and suggested complementing scales with qualitative feedback.

Our evidence from qualitative calendars shows the robot’s effect on anxiety and agitation as the second and third most common *reasons for robot use*, respectively, strengthening the suggestion that affordable robot pets can produce well-being outcomes. Furthermore, interviews and calendar free-text observations demonstrated that the robots were calming, reduced anxiety, improved mood, relaxed residents, reduced agitation, and provided reassurance. The calendars also demonstrated that the primary *reason for use* of the pets was entertainment, thus providing a meaningful activity. This is congruent with the significantly greater reduction in apathy from baseline to follow-up in the intervention group compared to the control group. The importance of meaningful activities for older adults in care homes cannot be overstated, impacting physical and mental well-being [41]. Reduced apathy and greater engagement in an activity creates an improvement in the quality of life. The calendar and interview data suggest that older adults cared for and nurtured robots, which perhaps provided a sense of responsibility and purpose. Although most nurturing seemed to involve cuddling and fussing the animals, there were also counts of residents feeding, dressing, and grooming the pets, thus providing care.

In contrast to prior work suggesting that robots could improve communication and interactions [42], our Holden communication scale results demonstrated no significant difference in communication as a result of robot implementation. However, our qualitative results suggested that robots encouraged communication, mediating social connection as shown in previous work with Paro [42]. The communication scale we selected provides a measure of resident speech and conversational ability [27], a possible limitation of our work. Future research may seek to employ measures of social cohesion and quality of interactions. Interestingly, our qualitative results did demonstrate evidence of speech and conversational ability improving in some instances, such as residents with severe aphasia showing no signs of the disease upon communicating with the pet dog. This is a profound result, although not replicated in the chosen scale, thus requiring further exploration in future research.

Our experience of sampling observations through calendars [30] also provided insights into the types of uses of the robots. As we did not provide an intervention dose, this aids in understanding the likely real-world use of the devices. The calendars demonstrated a range of uses, from short 15-minute sessions to 24/7 use by some residents who *adopted* the pets, keeping them day and night, until care staff retrieved them to be cleaned and shared. This result highlights a limitation of prior robot pet trials with highly controlled and prescribed intervention doses [7,29], as real-world use is likely more flexible and variable. Our results demonstrate that robots had high levels of use, and were clearly well adopted into daily practice. Observing staff reported evidence of residents loving pets and displaying ownership tendencies. Importantly, the study demonstrated no novelty effect for devices over 8 months, providing evidence against novelty as a concern for robot pet research and implementation [43]. Regarding use type, there were only 10 counts of group sessions recorded as the *reason for use*; however, these were all recorded prior to COVID-19 restrictions. Evidence in interviews after the 8-month study suggested that most robot use was on an individual basis. Previous work has varied in either group [2,3,5] or individual robot interventions [16,17,37]. While our work suggests that individual intervention is most common, we are unable to comment on the generalizability of this result to nonpandemic contexts. However, availability of multiple devices appears desirable owing to some issues in sharing and jealousies evidenced in our qualitative results.

The qualitative evidence also gave some further insights into robot design, based on longitudinal experience with robot pets. As in our previous work [10], stakeholders commented on hygiene as a design limitation of current devices, requesting removable shells for easier cleaning. Participants again supported the importance of realistic design, life-simulation features, and interactivity. Stakeholders felt the JfA cat had more appropriate vocalizations than the dog, although the importance of mute options (which the JfA devices have) was highlighted. Ultimately, design preferences seen here in the longitudinal work are consistent with the results of our previous cross-sectional design studies, supporting the validity of our earlier results [9-12]. In contrast to our previous work [14] suggesting that the devices were suitably robust, this study reported cases of breakage. We know of 5 broken pets throughout this trial, from a total of 18 pets (16 original and 2 replacements). One JfA cat had broken limbs (cause unknown), which did not hinder its use; 1 cat was dropped in urine, which rendered it unusable; and 3 dogs had technical malfunctions. The variance in reported robustness between prior work and this study could have resulted from the different settings (supported living vs care homes) and the thorough exploration with more devices, creating greater opportunity for issues to become evident. Despite the issues, only 2 devices required replacing as the other 3 remained mainly usable.

Owing to the timing of this trial, we were able to gather some understanding of the use and impact of robot pets during the COVID-19 pandemic, and resultant lockdown and isolation, which is entirely novel. The evidence suggests, in line with a previous report [44] and our suggestions, that homes took extra precautions for shared robot use. Despite this, the pets were highly valuable tools during the pandemic and lockdowns, with care staff reporting strongly on the value during the unprecedented times. The pets aided in reducing loneliness and providing company and comfort for residents experiencing long periods without visitors or usual excursions. The pets were also used for residents shielding in self-isolation, and were beneficial for those alone in their bedroom. This is a positive result and has implications for care homes and other aged care services, suggesting that the provision of robot pets for individual use during pandemic situations may ease the challenges of isolation. Isolation is particularly pertinent for care home residents [39], highlighting the value of this finding. Despite these benefits, the risk of use during pandemic situations must be thoroughly assessed, in light of the risks detailed in a previous report [44]. Here, our results demonstrated high numbers of mortalities in collaborating homes. While our enquiries suggested that the deaths were unrelated to robot presence, the risk needs to be considered appropriately, as with all shared surfaces, social contact, and cleaning procedures in the homes.

Regarding general acceptability, the summative impact question demonstrated that, encouragingly, 85% of residents who interacted with the robots experienced a positive impact based on carer observations and 74% of residents included in the analysis interacted with the robots. However, the finding that almost a quarter of the residents included in the analysis did not interact with the robots indicates that the devices lack universal appeal. This result, combined with 11 qualitative counts of robot dislike, is congruent with previous research reporting variation in the response to Paro [8,29], who was described as a “therapeutic tool that’s not for everybody” [45]. In contrast to the prior work with Paro, where acceptability was reported to be 50% [45], the JfA devices seem more generally acceptable.

Regarding device suitability, the results demonstrated that residents who interacted with the robots had, on average, more severe dementia, communication issues, and challenging behavior. Previous work has also suggested that companion robots were more suitable for individuals with dementia [44]. This could suggest cognitive impairment and dementia severity as predictive of likely robot acceptance and benefit; however, this contradicts our earlier work, which demonstrated robot pet acceptability among independent older adults [9] and care home residents without dementia [10]. It is possible that the impact of COVID-19 and the restriction on sharing robot pets in groups led to the prioritization of interactions for more impaired residents. In the qualitative data, evidence suggested that robots were most enjoyed by and beneficial to older adults who had dementia, and also those who were bed bound (due to mobility or illness), less socially engaged (due to dementia), or in isolation (due to COVID shielding). Additionally, residents who were disinterested in the robots were more socially engaged, preferring to play games and socialize with other people. While social engagement appears to be negatively correlated with dementia severity, the results may indicate that both dementia severity and social isolation predict the likelihood of accepting and benefiting from robot pet interventions. This could explain the acceptability of robot pets by more independent older people in prior work [9], as despite not having dementia, the older people lived in individual flats and reported requirement for social company. In previous work with independent older adults living in the community, 4 of 12 robots were rejected [16], with community-dwelling older people less vulnerable to isolation and loneliness [38]. Additionally, Pino et al [46] reported on healthy older adults feeling able to benefit from socially assistive robot (SAR) support, while Tkatch et al [20] reported positive benefits of JfA devices for *self-reported lonely individuals* despite them living in the community. Loneliness and dementia severity are thus likely to be predictive factors in the acceptance and benefit of robot pets in future implementations.

### Strengths and Limitations

A strength of this work is the pragmatic mixed-method approach. The use of calendars to support interviews and psychometrics allowed for ecologically valid appraisal of subjective experiences, yielding comprehensive views of activities that may be difficult to assess using cross-sectional questionnaires or interviews, which can cause memory strain and aggregation [30]. A second strength is the somewhat novel approach to this trial that did not specify an intervention dose. This allowed for ecological validity and assessment of the effects on resident well-being based on the likely real-world use of robot pets, with the intervention dose reflecting real-world circumstances. In this regard, our results demonstrate the effectiveness of robot pets and the impact robot pets may genuinely have with real-world implementation, rather than their efficacy, as well as the impact these robots have under highly controlled research contexts with specified intervention doses [32]. Furthermore, not defining an intervention dose avoided the ethical concerns of encouraging robot interaction when residents were resistant and removing robots when they were being enjoyed, as encountered previously [8]. One limitation of this work is the lack of participant responses to the loneliness measure, resulting in the inability to assess the impact on loneliness quantitatively. We had also originally intended to collect medication records, but due to the impact of the COVID-19 pandemic, this was not possible. Prior work with Paro has suggested resultant decreases in the use of psychoactive and analgesic mediation [7], thus this remains a topic for future research. A second limitation is that our analysis reports on the NPI subdomain scores, further to the NPI total, with previous work cautioning that while use of NPI subscales has been popular, validity and reliability are mainly established for the total measure, with the validity of individual scales requiring further testing [47]. A third methodological limitation results from the inability to blind collaborators to conditions. It is possible that the significantly improved outcome measures in the intervention group were a consequence of the inability to blind collaborators. This challenge has been reported in prior Paro RCTs, where the influence of participating in the research itself raised staff awareness to improvements and contributed toward positive findings [3]. It is not possible to distinguish this effect from the intervention. Thus, there is some possibility of positive reporting bias from our collaborators. Additionally, the inability of 2 care home staff to co-jointly complete the 4-month outcome measures may have reduced the validity of the 4-month scores.

The use of a cluster RCT may also be perceived as a limitation over standard RCTs [3]. However, research with older adults and in care home environments presents specific challenges, differing greatly from clinical environments or laboratories. Residents often have dementia, and the ability to randomize residents individually within homes to receive/not receive a robot intervention would be challenging and unethical. Creating clusters from care homes as units, rather than randomizing residents individually, allows for research such as this [3,29]. A final consideration is that the psychometric scales we selected were all designed and validated for older adults and those with dementia. Not all of our participants had dementia; however, the scales were deemed appropriate by our collaborators owing to the high prevalence of dementia in long-term care facilities, such as care homes [29]. Additionally, the content of the chosen scales appears appropriate for older adults with and without dementia, and even those without diagnosed dementia who sometimes experience onset symptoms. Indeed, very few of our participants received a very low score on the dementia severity scale.

### Conclusion

Our results suggest that affordable robot pets may have important well-being effects in older adults, including reduced neuropsychiatric symptoms (depression, delusions, elation, anxiety, and apathy), with qualitative accounts also supporting reductions in agitation. This work also suggests that robot use impacted occupational disruptiveness, as an indicator of care provider burden. The findings also support a no novelty effect for affordable robot pets and suggest that the best practice is the permanent availability of multiple devices. One key finding is the contribution to the discussion on the suitability of robot pets. Previous work has suggested that robots are best suited to residents with more severe dementia. This was supported in our work; however, we also suggest that subjective loneliness may be a predictive factor in the acceptance and benefit of robot pets. This work has also demonstrated the important value of the individual use of robot pets during the COVID-19 pandemic, with easing of the challenges of isolation through the provision of social companionship.

## Appendix

Multimedia Appendix 1 Variation from the planned stepped-wedge trial due to the COVID-19 pandemic.

Multimedia Appendix 2 Further analysis of deaths during the trial and the impact of COVID-19.

Multimedia Appendix 3 Histograms demonstrating normality issues for the primary outcome of neuropsychiatric symptoms at baseline and 4 months in both the intervention and control groups.

Multimedia Appendix 4 Full table of themes and evidence.

Multimedia Appendix 5 CONSORT 2010 checklist for Cluster Trials.

## Abbreviations

ITT: intention to treat
JfA: Joy for All
NPI: Neuropsychiatric Inventory
RCT: randomized controlled trial

## Data Availability

The data sets generated and analyzed during this study are available from the corresponding author on reasonable request.
