# Supplementary material for: Implementing Affordable Socially Assistive Pet Robots in Care Homes Before and During the COVID-19 Pandemic: Stratified Cluster Randomized Controlled Trial and Mixed Methods Study
Source: JMIR Aging. 2022 Aug 24;5(3):e38864. doi: 10.2196/38864 (PMC9407160; doi:10.2196/38864)
Supplement: Multimedia Appendix 1 [file aging_v5i3e38864_app1.docx]

**Multimedia Appendix 1.** Variation from the planned stepped-wedge trial due to the COVID-19 pandemic.


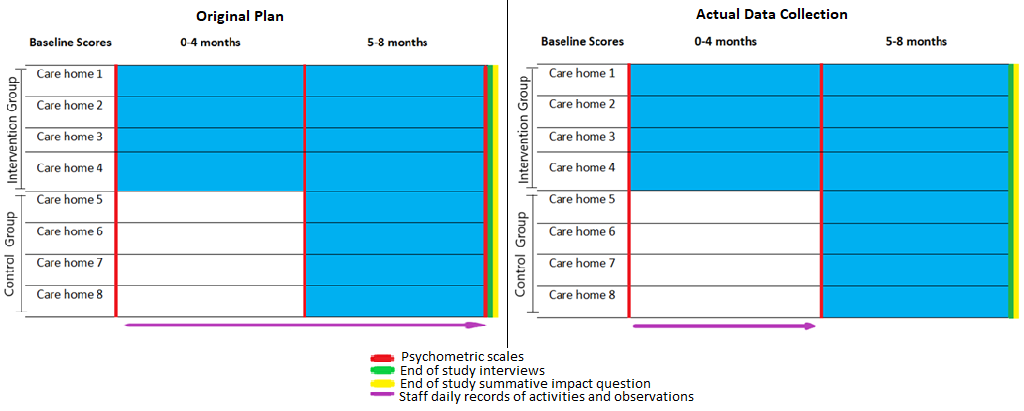


Figure S1**:** Stepped wedge study design as originally planned (left), study design as carried out as a result of the pandemic, showing the removal of quantitative data collection at 8 months and the ending of staff diaries at 4 months (right).

Blue shading represents exposure to the intervention (availability of robopets), whilst the white area represents the control phase to receive usual care. As demonstrated in Figure 1, the quantitative scales represent a parallel control trial, where metrics are collected for residents in the control group and intervention group at baseline and following four months. As care staff capacity was limited by the pandemic, scales were not repeated at eight months. Diary records were maintained in both the control homes and intervention homes for the first four months. Due to limited staff capacity during pressures of the pandemic, diary entries were not recorded from four to eight months. The qualitative impact of robots for all residents in all eight homes was collected at eight months through telephone interviews and a summative impact question.
