# Supplementary material for: Implementing Affordable Socially Assistive Pet Robots in Care Homes Before and During the COVID-19 Pandemic: Stratified Cluster Randomized Controlled Trial and Mixed Methods Study
Source: JMIR Aging. 2022 Aug 24;5(3):e38864. doi: 10.2196/38864 (PMC9407160; doi:10.2196/38864)
Supplement: Multimedia Appendix 2 [file aging_v5i3e38864_app2.docx]

**Multimedia Appendix 2.** Further analysis of deaths during the trial and the impact of COVID-19.

In conversation with the care homes at 8 months, three of the collaborating homes reported Covid outbreaks (Home 1, 3 and 5), although at different times. Despite the high number of deaths in Home 1, collaborators reported Covid was not present on death certificates of participating residents, but this does not mean Covid was not present considering issues in testing early in the pandemic. For a better understanding of deaths in control and intervention homes, Table 1 displays deaths among residents in the trial homes that were not consented to the trial.

Table S1: Resident deaths in participating care homes from baseline to follow-up

|  | | **Not consented** | | | **Consented** | | | **All** |
| --- | --- | --- | --- | --- | --- | --- | --- | --- |
| **Care Home** | **Total residents** | **Total** | **Survived** | **Died** | **Total** | **Survived** | **Died** | **Total Died** |
| **1** | 33 | 24 | 16 | 8 | 9 | 3 | 6 | 14 |
| **2** | 16 | 5 | 4 | 1 | 11 | 10 | 1 | 2 |
| **3** | 36 | 27 | missing | missing | 9 | 4 | 5 | - |
| **4** | 36 | 24 | 17 | 4 | 12 | 9 | 3 | 7 |
| **Total** | 121 | 80 |  |  | 41 | 26 | 15 |  |
| **5** | 36 | 29 | 13 | 16 | 7 | 4 | 3 | 19 |
| **6** | 27 | 14 | 10 | 4 | 13 | 12 | 1 | 5 |
| **7** | 31 | 18 | missing | missing | 13 | 12 | 1 | - |
| **8** | 38 | 29 | 19 | 10 | 9 | 9 | 0 | 10 |
| **Total** | 132 | 90 |  |  | 42 | 37 | 5 |  |

The total number of deaths in the eight homes is comparable between the control and intervention group. Of note, Home 1 has two separate units, a dementia unit and general unit. The general unit is housed in a separate building, although attached to the dementia unit. The dementia specific unit was the cluster in this trial, referred to as ‘Home 1,’ with 33 residents in total. The residents in the two units do not interact, and robots were not shared with residents in the general unit. To this regard, the units are comparable in location, size and management. In the general unit, 17/33 residents died during the four-month study period, and had no interaction with robots. This is comparable with 14/33 in the dementia unit, which would suggest the care environments in general were greater contributors to viral spread than robots.

During the early stages of the pandemic, care homes suffered documented shortfalls in personal protective equipment and testing. Care homes also received Covid positive residents discharged from hospital. The three homes that experienced an outbreak are additionally all nursing homes, with a high concentration of vulnerable individuals, further to a great number of shared surfaces and fomites and direct contact between residents. Care home residents were not socially distancing from each other. Covid-19 is more likely to be transmitted as aerosol than surface transmission. Thus, the care environment itself is particularly vulnerable to viral transmission, and it appears likely higher mortality in the intervention group relates to unfortunate timing of Covid outbreaks, and particular residents consented for the research (Table 1).
