# Supplementary material for: Implementing Affordable Socially Assistive Pet Robots in Care Homes Before and During the COVID-19 Pandemic: Stratified Cluster Randomized Controlled Trial and Mixed Methods Study
Source: JMIR Aging. 2022 Aug 24;5(3):e38864. doi: 10.2196/38864 (PMC9407160; doi:10.2196/38864)
Supplement: Multimedia Appendix 3 [file aging_v5i3e38864_app3.docx]

**Multimedia Appendix 3.** Histograms demonstrating normality issues for the primary outcome of neuropsychiatric symptoms at baseline and 4 months in both the intervention and control groups.


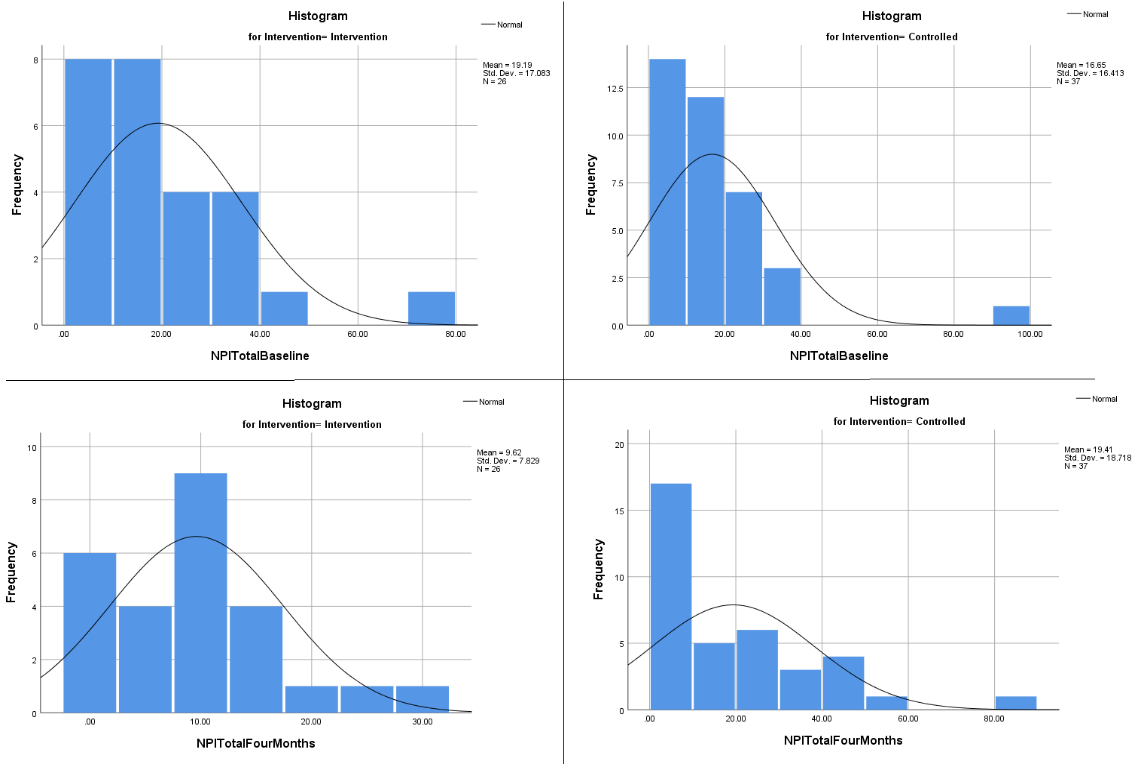


Figure S1: Normality histograms for primary outcome (NPI total) at baseline (top) and four months (bottom) for intervention (left) and control (right) groups
