# Supplementary material for: Implementing Affordable Socially Assistive Pet Robots in Care Homes Before and During the COVID-19 Pandemic: Stratified Cluster Randomized Controlled Trial and Mixed Methods Study
Source: JMIR Aging. 2022 Aug 24;5(3):e38864. doi: 10.2196/38864 (PMC9407160; doi:10.2196/38864)
Supplement: Multimedia Appendix 4 [file aging_v5i3e38864_app4.docx]

**Multimedia Appendix 4.** Full table of themes and evidence, followed by narrative of analysis

| **Theme** | **Codes**  **(n in interviews) [n in diaries]** | **Example Evidence** |
| --- | --- | --- |
| Adoption | Love (11)[13] | “he loved it. It was it was almost emotional watching her, react, and respond to it” (Interview_home4),  “It's how soft it is, the long, the long fur on the cat. How pretty the faces. It's just. Yeah, she loves it.” (Interview_home5),  “[Name] loved the cat” (Interview_home2),  “She loves him sooo much and wants him all the time” (Calendar_home2)  “[Resident] loved the cat today, was smiling” (Calendar_home3)  “[Resident] as always, loved the dog, kept the cat in her room last night” (Calendar_home2)  “[Resident] was chatting and stroking the dog, she loved the dog as if it was her own” (Calendar_home4)  “[Resident] loves it and goes to bed with it” (Calendar_home2) |
|  | Ownership (18)[6] | “Some thought they were actually their real pet. claim to take possession of them, that they were theirs and we had to sneak them away.” (Interview_home1),  “[Name] loved that cat, it was her cat, she would look after it” (Interview_home2)  “It's very much ‘his’ really. He's really, we couldn’t really part him from it. It’s offered him a lot of comfort.” (Interview_home8)  “completely and utterly adopted by we had two pets and they were adopted by two residents and throughout so, one of my favourite things is when one of the residents goes to her room, and the cat goes with her and it's just sort of gives her a focus.” (Interview_home5) |
|  | Individual use (9)[14] | “If you've got a pet, it's yours. Ownership seemed, you know, one of the points of it” (Interview_home5)  “Seeing […] nonplussed reaction of other [residents] is like, well, we don't need to circulate it around. It's useful for certain people, so it's no good sort of having it as a house pet” (Interview_home5)  “mostly individual To be honest, they mostly went round individually, to begin with there was a few group sessions to introduce them and everything. But most of the people who benefited most were the ones that were in their rooms all the time. Or weren’t particularly having conversations with other residents or anything, with dementia, and were past the group stage and are better on a one to one.” (Interview_home1),  “Individual, it's like having, if you’ve got your own pets. So you like having sort of one to one with the pets. Talk to them” (Interview_home2)  “better if they’re in their rooms with people individually, easier to manage because nobody else can see them” (Interview_home6)  “Had down in own room for whole day, enjoyed cuddles” (Calendar_home1)  “[Resident] keeps it in her bag and gets it out when upset” (Calendar_home3) |
|  | High level of usage [12] | “[Resident] has kept the dog all day” (Calendar_home1)  “[Resident] still has the dog, all day” (Calendar_home1)  “[Resident] really loved the dog, left her with it because she didn’t want to let it go” (Calendar_home2)  “[Resident] loves it and goes to bed with it” (Calendar_home2) |
|  | Jealousies or possessiveness (6)[6] | “She doesn’t like to give it back really. She doesn't know that it's not a real cat. However, we can't really let her, we can let her have it for an hour or so. But we have to get it back off. Can be quite challenging, she does love it though” (Interview_home7),  “Yeah, some people get sort of quite attached to them. […] they won’t leave them go” (Interview_home6)  “I think he would probably a bit angry if we moved and get, gave it to someone else.” (Interview_home8)  “[Resident] seems to dominate the cat if not careful” (Calendar_home2)  “[Resident] chatted to cat, become unsettled when cat was taken away for tea time and is reporting us for animal cruelty” (Calendar_home4)  “[Resident] chatted to the cat but wouldn’t share” (Calendar_home4)  “[Resident] loves the dog and will not let anyone else interact with it” (Calendar_home3)  “[Resident] has his own dog, but likes to round them all up often” (Calendar_home2)  “a brief moment of oh I wish I could have it on my lap, but oh no don’t bother.” (Interview_home5) |
|  | No novelty (9) | “This morning she stroked the cat like it was, you know, like she's done and loved it from day one.” (Interview_home5),  “Yeah, the cat, she looks at it. You can see the love in her eyes, every day. When she stroked it this morning, there's no change in how much he adores it. It's so lovely to see.” (Interview_home5)  “I think it’s only changed in the sense that it's been adopted. Yeah. So the ones that we've had I don’t think people have got bored of them.” (Interview_home8)  “It's a continuous type type of thing. Yeah. Yeah. And, realistically, if people would get bored of it, if you put it away for two weeks bring back out, most people might not remember.” (Interview_home8)  “No no I don’t think they’re less, I don’t think they’re bored with them, They’re just as useful and I think, residents are just as interested now from where to start, I don't think much change in my opinion. I don't think that's changed. You know, we've certainly with my use when I get them out.” (Interview_home7)  “I would say it hasn’t change, they’re just as interested in them as they ever were” (Interview_home7) |
|  | Naming (7) | “One gentleman basically adopted the cat, and named him” (Interview_home8)  “She's she sees him from the hallway, which is what, […] 10 meters away, she can see him and she's gone oh hello jack. Oh, there he is. And she knows he’s there and is meant to be there. And she likes it when he is there.” (Interview_home5)  “The dog was called Ben by this lady, and had to come to the hairdressers with her […] He’s Ben isn’t he and so he shall be forevermore unless somebody else decides to name something else.” (Interview_home2)  “We did have a little sort of competition about what to call him, but actually as times gone on and people have come and gone. They just made him a dog that they had right. ” (Interview_home6) |
|  | Group sessions [5] | “Enjoyed cuddles in group session” (Calendar_home1)  “Cat was enjoyed by all residents in the group, it brought a smile to their faces and all engaged in stroking its fur” (Calendar_home2) |
|  | Personalising (1) | “It’s ended up with a little pink bow in his hair. It went into her room without one and when it came out with his pink bow on and everyone loves it and it’s just stayed on there. She strokes it and tickles it under the chin. She smiles at it. It is wonderful.” (Interview_home5), |
| Wellbeing effects, particularly mood | Calming (10)[20] | “it does calm him down, he has made an attachment to it, and he's named it. And that continued, even with his dementia.” (Interview_home8)  “The best way to describe it is a lot more emotional. Yeah. A more emotional connection. I mean, that that was quite generally with most people that used it. Yeah. And it definitely had a calming effect.” (Interview_home8)  “She would respond really well. She would almost think that they're real and really, really calming effect on her, for that instance, is really, really, really effective.” (Interview_home4)  “Family […] they realized how much he aided her and how much it calmed her down.” (Interview_home1)  “he's able to just to just sort of calm calm himself really, just through stroking the dog, and he'll talk to it, you know, he'll sit at the window with it, on the table, he’s got a table for him, we'll put it on the table for him and, he’ll sit looking at the garden and stroke the dog, and it really does have a positive calming effect on him. On his mood. So we can use we can use them for the escalation. And residents that are anxious and it might actually prevent them from from getting any, any worse, Yeah, it will calm them down and help distract them from having a bit of a meltdown, for want of a better word” (Interview_home7)  “Enjoyed sitting and cuddling the dog, calmed down” (Calendar_home1)  “Calmed her down in a moment of need” (Calendar_home1) |
|  | Enjoyment (1)[19] | “Yes it’s been brilliant, brilliant. A lot of them are really really keen on them. Really enjoyed having them, some thinking they were real, some realizing they weren't but enjoyed petting them.” (Interview_home1)  “[Resident] enjoys the cats company” (Calendar_home4)  “[Resident] enjoyed the cat, spoke to it” (Calendar_home3)  “[Resident] enjoyed looking after the cat” (Calendar_home2) |
|  | Anxiety reduced (3)[13] | “She passed on bless her. We had one particular lady that it worked for every single time, it lowered her anxiety. ” (Interview_home4)  “[Resident] enjoys the dog and helps reduce anxiety and agitation” (Calendar_home4)  “[Resident] missing own dog, loved the cuddle, less stressed” (Calendar_home1)  “[Resident] became unsettled with another resident, sat with her dog and fell asleep” (Calendar_home4)  “[Resident] appeared to really relax and de-escalate anxiety” (Calendar_home4)  “Helped to reduce agitation and anxiety” (Calendar_home2) |
|  | Companionship (7)[6] | “They love the companionship, they you know, they thought it was beneficial as a human talking to them.” (Interview_home1),  “New resident was unsettles, she has spent the afternoon with the cat, she said she knows it’s not real but enjoys it’s company” (Calendar_home1)  “[Resident] enjoys cats company” (Calendar_home4)  “[Resident] enjoys company of both to distract him” (Calendar_home4) |
|  | Smiles and happiness (1)[9] | “She smiles at it. It is wonderful.” (Interview_home7)  “[Resident] was very happy to see dog and talking and petting it” (Calendar_home1)  “[Resident] smiled stroking the cat, “you’re lovely” (Calendar_home4)  “[Resident] talked to the dog, lots of smiles” (Calendar_home4) |
|  | Engaging resident (10) | “Yeah, both, have a good old chat, try feeding them, urm. They do interact with them as though. Especially the cat again. A though It was a real cat. Yeah. They get told they're naughty boys (laughs)” (Interview_home8)  “Yeah. And I think it's quite handy when, they’re sat in the room, because then they're turning on itself, is again, that's another activity which you can instantly engage with. And then look for it and go oh what’s that noise, blah bah blah, It's not just the case of sitting down and stroking it. There are other ways it can be used” (Interview_home8)  “I've actually got a cat in my office that sat on my shelf here, and I have residents that come in, to come in and talk to the cat. They always come and say hello” (Interview_home4)  “The one in my office now, although it’s out of action it’s not going out to anybody, they’ll come in and just talk to it” (Interview_home4)  “But more interactive. Not falling asleep or whatever, instead she was interacting with the dog and with other people about the dog.” (Interview_home2)  “perhaps that’s where the cat goes wrong. It doesn't it does. Most things like the rolling the meowing and the purring and you know, like, like a cat would. But with a dog. I think it's a little bit more engaging, you know, a bit more. Like it's looking at you like, like it’s understanding you. Yeah.” (Interview_home2) |
|  | Relaxing or settling [7] | “Enjoyed sitting with the cat, helped relax him” (Calendar_home4)  “[Resident] relaxed and enjoyed” (Calendar_home1)  “Relaxed for a while, calmed down” (Calendar_home1) |
|  | Mood improved (7) | “Because of COVID, obviously, we're not we're not able to do that. So their residents, and we do have to recognize that they are missing out on having their own pets. I feel it's been a God send really having them. Especially to be able to de-escalate, for certain residents, it’s been very helpful having them, really” (Interview_home7)  “Mood, definitely the moods. Yeah, it lifted quite a few of their moods.” (Interview_home6)  “They certainly lift spirits, that's for sure.” (Interview_home6) |
|  | Provides a focus (5) | “One of the residents goes to her room, and the cat goes with her and it's just sort of gives her a focus.” (Interview_home5) |
|  | Distraction (3)[2] | “You can use it as a distraction. Okay, so it kind of takes away from that feeling. Yeah, yeah. You can use it as a distraction. You can engage in him in a different way to kind of totally avoiding the anger building up.” (Interview_home8)  “The ones who have dementia that tend to get some the mood swings. Yeah, we’ve got one now who can have a lot of mood swings, as she knows they're not real. And she will take it which is more of a distraction. And it will distract her for a while.” (Interview_home1)  “Yeah, it will calm them down and help distract them from having a bit of a meltdown, for want of a better word” (Interview_home7) |
|  | Agitation reduced [5] | “Seemed to ease [Resident’s] agitation” (Calendar_home2)  “[Resident] was feeling very agitated, sat with the dog in lounge and it really calmed her down” (Calendar_home4)  “[Resident] really enjoys dog and reduced agitation” (Calendar_home2) |
|  | Entertainment and laughter (1)[3] | “I think the dog would probably be more entertainment, because you're kind of trying to get people to use it more, but the cat was definitely along the lines of the interaction?” (Interview_home8)  “[Resident] laughed at the dog because she said something and dog barked as it’s response” (Calendar_home4) |
|  | Therapeutic (3) | “Because of the covid the chairs are spread apart. And because of whatever reason, a slightly bigger table was put between the owner and her friend. And the cat just lies between, you know, almost sort of elbow to hand like a long the arm of the chair. It is always there and if is pushed back a bit I bring it forward because she’s stroking it everyday regularly. Yeah. So that's nice, isn't it? That's one of the therapeutic things about pets, it the touch when you’re stroking it.” (Interview_home5) |
|  | Reassurance (3) | “Yeah. I would say the majority. Yes. They [staff] have found a tool for giving comfort, reassurance. That kind of interaction, and starting interaction as well. Using it as a topic.” (Interview_home8)  “I think it's [heartbeat] reassuring. Okay. That that was like sort of thud thud thud, that you get with it with a heart. I think that would be quite reassuring. I mean, they did like, yeah, they did like the purring with the cat. Yeah. As well. And that was louder, but the trouble with the cat, his bodies hard.” (Interview_home2) |
|  | Sundowner, (2) | “She was a Sundowner as well as a particular time of day where she would become more anxious. And we would know actually, if we get our cat or dog then she would instantly calm, really, really effective for that particular person.” (Interview_home4) |
|  | Reduced boredom (1)[1] | “But it’s there if they want it, need it, yeah. They’re upset, they’re bored, give them the dog.” (Interview_home2) |
|  | Enabled eating [1] | “Calms her down and makes her eat by sharing with the dog” (Calendar_home3) |
| Effects on Communication | Communication with pet [25] | “[Resident] loves to chat to cat” (Calendar_home4)  “[Resident] sat stroking the cat and talking to it, wrapped it up in a blanket” (Calendar_home2)  “[Resident] very fond of cat, chatted, unsettled now content” (Calendar_home4) |
|  | Communication with others, and speech (19) [2] | “You know, I like to walk into it. It responds when it moves. It also gives the staff and other residents a reason to talk to them. It's almost like bringing a bit more interaction between them and the residents. Like as they're talking about the dog. You go for a walk in the park, you don't talk to people who are walking you talk to people who have got dogs don’t you, you talk to them about their dogs.” (Interview_home5)  “You know, it must be it must really focus them. And they because we do have several residents with speech, they are able to talk very well, but it's completely jumbled. And it's really difficult to make sense on time, what they’re saying. However, when you put the animal in front of them, and another lady that has had greyhounds, and she loves dogs, and you know, when you give them the pet, then they come out with several very, very clear sentences. So that's quite critical. Really.” (Interview_home7)  “Yeah. I would say the majority. Yes. They have found a tool for giving comfort, reassurance. That kind of interaction, and starting interaction as well. Using it as a topic.” (Interview_home8)  “Yeah. Gives you something to discuss. As well, which sometimes can be quite difficult. For some staff I think” (Interview_home8)  “Now we had group sessions on our planner, we have a planner every week. So I could plan for a week it was it was planned to have pet therapy, and it engaged conversations and that about pets that they used to have or what they remember about, there's not just engagement with the animals, it’s also reminiscing about the past events as well, which is quite good and a group activity” (Interview_home4)  “the positive effects. Sometimes her speech is really quite muddled. However, when you put the cat in front of her, as you can see on this little video, her speech becomes very clear as she talks to it” (Interview_home7)  “But certainly, the staff, will take one of the pets, take one out and spend time, you know, so they're interacting, they don't know, they're interacting more. They might it might spark conversation about about the residents pet or just generally their own the staff members own pet.” (Interview_home7)  “It would appear to me that one of our ladies who has quite severe expressive aphasia, when engaging solely with the dog shows no signs of this and communicates clearly with it, I wonder if this is because, similar to music it comes from the emotional part of the brain. ” (Interview_home3)  “I have also sent a photo of two ladies who usually spend their day in conflict with each other. The picture I think speaks for itself. [picture shows two older ladies sat on the same armchair, smiling/laughing and looking at the dog]. The response has even surprised an old cynic like me.” (Interview_home3)  “[Resident] adores the dog, vocal conversation point” (Calendar_home4)  “Group interaction, [Resident] initiated conversation” (Calendar_home2)  “I gave her the robot cat to stroke and left her with it, she was then cuddling it and interacting with another resident and their family” (Calendar_home1) |
|  | Reminiscence (5)[1] | “It is, it is very much so. And then you can get talking about their dog. Or the other dogs, and all that sort of thing. Yeah, it is very much reminiscence because that's what they see as their dog. This is them, this is my dog” (Interview_home2)  “it engaged conversations and that about pets that they used to have or what they remember about, there's not just engagement with the animals, it’s also reminiscing about the past events as well, which is quite good and a group activity” (Interview_home4)  “That gets them to talk about something that's joyful if you know something that they remember with joy rather than. Yeah, hopefully they wouldn't remember that it died. Yeah.” (Interview_home2)  “[Resident] talked about his own pets, reminiscence of dog” (Calendar_home4) |
|  | Interaction (4) | “Ben [dog] enjoyed it for hairdressers because it's lots of noise going on, so he kept turning his head, even the staff like him!” (Interview_home2)  “Talk to them and the fact that the dog will turn as well. I don't know, does it respond to voices or is that just my imagination?” (Interview_home2)  “Because it will look at you when you're talking if someone if someone comes along and talk then it’ll move, and that appears to be good, and that's obviously what it was” (Interview_home2) |
| Isolation and Covid | Covid use (15) | “it's gone fantastically. And I'm really glad we have them especially at this ridiculous time. Yeah. I couldn't have thought of a better time for us to have them.” (Interview_home5)  “I find that since we've had the covid situation, we're not actually allowed to have any real animals in the nursing home, we have, we do have two pet cats here. But since we're not allowed to have real life dogs in, they've come in really, really useful. Really useful thing to have. ” (Interview_home7)  “Yeah, I think because obviously with covid, it was offering comfort. That little bit of social interaction to get, referring to the gentleman adopted, he doesn't really interact very well with other residents. And he can become quite angry. Okay. So yeah, it’s given him that. That relationship. If that makes sense. Yeah. He's got his friend. Yeah. And, yeah, before we’ve obviously been conducting video calls, etc. Yeah. It has offered that comfort and I guess a little bit of a distraction as well.” (Interview_home8)  “That's the whole thing, I just I was overjoyed that it happened at that time. Due to that reason [no family visits]. Definitely.” (Interview_home5)  “Well, who knows what these two would have been like, during lockdown without them. But I feel 100% that they have improved the situation. Yeah. From the point of view of Yes. Company, yes a focus, They can see and think oh yeah and. Remembering even though dementia requires or doesn't allow you to remember, there is definitely recognition. Oh, yes. I know, I'm in the right place, because this is sat next to me.” (Interview_home5)  “Yes I would say so yeah. Because they're not seeing their relatives. Yeah. So especially the very beginning. But now we've got a screen up and some relatives one at a time can come in for half an hour. At the beginning. There wasn't anybody you just in maybe FaceTime or on the telephone. But yeah, so definitely useful. Yeah, very good.” (Interview_home6) |
|  | Cleanliness and infection control (9) | “Those who adopted it and then COVID came in. So it was a case of well to reduce the risk of germs spreading, that it’s best that they stay with one person” (Interview_home5)  “During COVID, etc, we’ve got to be more vigilant about cross contamination. And they are quite difficult to keep clean. And the cat. Yeah, we did have a lady that enjoyed feeding it. ” (Interview_home8) |
|  | Isolation (5) | “most of the people who benefited most were the ones that were in their rooms all the time. Or weren’t particularly having conversations with other residents or anything, with dementia, and were past the group stage and are better on a one to one.” (Interview_home1)  “the ones who find them most beneficial, are the ones that don't really come out their room. Or don't really socially interact, integrate, they’re more things that are more really useful for people that are, you know, not really interacting with anything else? ” (Interview_home1)  “But actually got, we used one when we had a lady in isolation, which is in her room now, because obviously, we're in that period, where she's kind of had that to herself for the whole week. And that's been really helpful in her isolation period as well.” (Interview_home4)  “She's not having that engagement with other residents. What do we, what can we help her to pass the time, she likes colour but you can only colour for so many hours a day, and yet we put the dog up there, and she liked the dog anyway, before isolation. So we knew it was going to be a winner.” (Interview_home4) |
| Design | Improvements (11) | “I think that this sounds really awful. I know, I know what response I’m going to get from the young lady next to me. She's had lunch. It'd be quite good. If we could always take the skin off (laughs) and wash it or replace it” (Interview_home8)  “I think because I think they could feel a little bit more weighted. Yeah. as well. Because obviously, that provides quite a lot of comfort for. People with lots of different needs. Because a few years ago, we had a baby doll. Okay. And they were much more successful if they had a realistic weight to them.” (Interview_home8)  “So basically, skin it and make it fatter” (Interview_home8)  “Yeah I think the actions and everything are sufficient, they don’t need to be too over the top. Yeah. And the dog is a bit difficult. I think if it didn't look so much like a puppy. Look, maybe like a small dog. Yeah. A small older dog, maybe? Yeah, yeah. Yes, the sizes are, like you say, wouldn't normally put a dog on the table would you” (Interview_home8)  “We took off the scarf because it was being you know, when the dog had a little scarf, and it's a little red scarf. Yeah, we found that the owner was doing, not necessarily good things with that. So I took it away as being either a choke risk or strangulation risk or whatever. Okay. So that's been removed. But apart from that, no, I some of the realistic things on the cat like the paws are just wonderful. It's been so well made. The cat is wonderful.” (Interview_home5)  “Just the weight and the way it sits on their lap. Yeah. Quite. quite important. I think.” (Interview_home1)  “Obviously, we've tried to keep them avoid using them around mealtimes and things, you know, to try to keep them clean. And they are washable. But the skin, the skin, tut, the coat doesn’t actually, it's not fully removable. From that point of view, maybe, you know, that can be a little bit challenging, which are so careful with them, you know, and I think if you, if we treat them respectfully, as if they if they were real, treat them with a bit of respect, and make sure that when the residents are eating or drinking, that they're not in their hands. You know we used to have a dear lady that used to feed teddy bears. You know, but they were washable Teddy Bear, obviously, our robot pets aren’t washable” (Interview_home7)  “So I think the dog is perhaps a little bit sturdier. Because it hasn’t got moving parts only, like heads and tail wags? Yeah, but it's not so many moving parts.” (Interview_home2)  “the only thing that we found, and I spoke to you about before about, this is, the heartbeat seems very, very quiet. It’s got a heartbeat but nobody can actually hear it. When you’re holding it, you can’t feel it.” (Interview_home2)  “[The cat] is not squishy enough” (Interview_home2) |
|  | Realistic (9) | “[The dog’s] not realistic. Because we've actually got larger ones now. Yeah. And I think they've been better, okay. I mean, there's ones we’ve managed to pick up on or something. And the functions aren't perhaps, as good. But I think because of the size and the features, people are a lot more happy for it to just be next to them, if that makes sense. Yeah, it's probably a bit more.” (Interview_home8)  “I mean, they bark and everything else. Make funny noises. And that, but the ones we've got here have got quite a bit of wear and tear, through their little lives. So, I'm not sure if it's like the how realistic it is. But I think it’s definitely got something to do with it. Whether it's the size? I'm not sure. Because obviously the cat you're able to put it onto people's laps onto the armchair, chair or the table next to them or something. And it's kind of normal cat behaviour, isn't it? Yeah. Where as the dog? If you set that on the table? It kind of doesn’t, not a dog” (Interview_home8)  “I think the turning and moving the head was very good. It made them look, you know that they were more realistic. Yeah. The cat lifting its head up to be tickled and rolling over onto his back to be tickled like a real cat would. Yeah, there was a bit more interactive and the dog for that reason, you know and so that was good. That's why I think most took to the cat more than the dog. We have got one that adores the dog, and he feeds it.” (Interview_home1)  “I mean, the dog to me, it just looks like a soft toy. Yeah. And I mean, the poor cat has got two broken legs. Good job it’s not real!” (Interview_home2) |
|  | Sound off (8) | “She puts up with it for so long and talks to it for so long and then she gets fed up with it, because every time you move, it sort of makes a noise doesn’t it. Whining or barking? She's trying to sleep and it’s barking!” (Interview_home1)  “I think the cat was more favorable than the dog. But I think that's just because it's a little bit quieter. We had to turn the volume off on the dog a few times.” (Interview_home4)  “But yeah, I mean, they're just annoying sometimes because obviously there they are. sensors aren't they if you walk past it, and it's a sound somebody's lap and then it's all of a sudden, meow. Yeah. I think the cat, the cat was better than the dog.” (Interview_home4)  “We have one lady, quite poorly. And she's still really obsessed with the dog makes its way up there. They're not always wanting the noise on though so there has been that.” (Interview_home1)  “You know, maybe the cat makes a bit of noise when it’s moving, to make a little bit less noise? I think that’s probably unavoidable. I think they're quite realistic to be honest. The cat meowing and the dog barking isn’t as realistic but I think they’re pretty good really, yeah I think they’re pretty good” (Interview_home7)  “It could be irritating. Maybe. Maybe if you turned the cat off, maybe that would be better. Or you can mute the mute button, I think.” (Interview_home2) |
|  | Expectations (8) | “Because obviously the cat you're able to put it onto people's laps onto the armchair, chair or the table next to them or something. And it's kind of normal cat behaviour, isn't it? Yeah. Where as the dog? If you set that on the table? It kind of doesn’t, not a dog” (Interview_home8)  “Exactly, yeah, and I think it’s that. Maybe to do with the size again, to use it. Because you said then, ‘puppy’ and a puppy wouldn't normally sit still at all! And well, yeah, yeah, whereas an older dog will” (Interview_home8)  “But especially when you compare the two dogs, together? The large one is more successful. Definitely. But again, as I said, it’s probably reinforcing that realism,” (Interview_home8)  “The dog is a bit difficult. I think if it didn't look so much like a puppy. Look, maybe like a small dog. Yeah. A small older dog, maybe? Yeah, yeah. Yes, the sizes are, like you say, wouldn't normally put a dog on the table would you” (Interview_home8)  “Well, actually, regularly she gently puts it on the floor. Okay, she either pulls it onto her lap and hugs it or puts it on the floor, because that's where she expects a dog to be to be.” (Interview_home5)  “Exactly. She puts it down by her ankle and then pats it on the floor. Yeah.” (Interview_home5)  “I think what happens is the cat meows all the time. Maybe that’s what it is, the dog does a few barks, but they're nice. When a cat meows, you've actually done something wrong.” (Interview_home2) |
|  | Weight and size (7) | “And it's not so heavy because heavy can be a thing. A lot of them are sorta quite slim built by then. And yeah, they are annoying after a while the weight on their legs. The dogs got that disadvantage. It's heavier. Yeah. And it's not so easily sat on someone if you know because it’s sat upright. More difficult. Yeah, it's more like got to sit by your side or if it's on your bed, but that doesn't quite fit. So well. As the cat.” (Interview_home1)  “I think the cat sits a bit more nicely on your lap if you're not mobile. Yeah, the dogs a bit heavier.” (Interview_home1)  “Yeah, just the weight and the way it sits on their lap. Yeah. Quite. quite important. I think.” (Interview_home1) |
|  | Breakage (7) | “We changed this battery obviously and then he just stopped working. So [manager] purchased another one and then the one we've got now has got a problem in it. So really, […] this replacement one makes a beep just to completely random beep instead of barking or instead of I mean the cat meows and purrs I think the dog just barks, but it beeps and then it's Click, click, click, click, click. So yeah, we're in a problem period at the moment with a dog but the cat is wonderful.” (Interview_home5)  “Yeah, yeah. I mean, I mean, the dog to me, it just looks like a soft toy. Yeah. And I mean, the poor cat has got two broken legs. Good job it’s not real!” (Interview_home2) |
|  | Battery life (4) | “Yeah, actually, battery wise they weren’t too bad actually” (Interview_home8)  “And the batteries didn’t last very long at all. We had to keep changing them, we had to buy the batteries and keep changing them. Because they were used so much.” (Interview_home4)  “the batteries were pretty substantial actually. I think we only ever changed them like once they were quite good.” (Interview_home1) |
|  | Importance of movement (4) | “one of the main things, I guess I appreciated when the first one went wrong, it was just turned off and left next to its owner. she interacted with it much less, so it kind of reinforced the fact the moving and the, well is just the moving as we don't we don't have it barking ever. So it's just a moving and the blinking and turning its head she talks to it because it's doing that. So that's much less and responded much less to it when it stropped moving so that’s quite important, it's quite important.” (Interview_home5)  “when it broke, is that it was she sort of lost interest sort of started to ignore it almost when it didn't move, it was amazing to watch” (Interview_home5) |
|  | Purring as relaxing (2)[2] | “And the purring as well. It's quite soothing, isn't it? Particularly with the lady who really benefited, she would sit and just stroke the cat and that would obviously start the cat purring and that’s relaxing in itself isn’t it.” (Interview_home4)  “They did like the purring with the cat” (Interview_home2)  “Liked the purring of the cat, relaxing” (Calendar_home2) |
|  | Heartbeat enjoyable (1)[2] | “That that was like sort of thud thud thud, that you get with it with a heart. I think that would be quite reassuring.” (Interview_home2)  “[Resident] loved the cat and dog and felt the heartbeat” (Calendar_home2) |
| Suitability | Dementia severity (31) | “I think realistically, the cat was, urm a lot more accepted than the dog and the dog seem to be useful for people further along.” (Interview_home8)  “You know, with retaining things with retaining things, you know, with regards to their memory, they're very much in the moment you know, we try to be Stepping into that moment and being in that world, you know, in that bubble that they're in, I guess, perhaps they don't have too much of that. Memories or whatever, you know, each time that they see the pet it’s quite new for them […] and that’s what’s lovely, they will never grow tired of them” (Interview_home7)  “I mean, I can say it does depend on where they are in their journey with dementia, etc. Yeah. But then they do believe the cat is real. Yeah, certainly the gentleman that's adopted. There may be moments where he thinks, oh it’s not actually real, but 90% of the time when interacting, he believes it to be real.” (Interview_home8)  “I'm we, most of our residents here have a dementia. varying levels. But yeah, but yeah the more advanced dementia, residents respond better to it.” (Interview_home4)  “But most of the people who benefited most were the ones that were in their rooms all the time. Or weren’t particularly having conversations with other residents or anything, with dementia, and were past the group stage and are better on a one to one.” (Interview_home1)  “the ones that haven't got dementia are still really with it, and they aren’t that interested in them” (Interview_home1)  “Possibly because they would just see as a toy as it is. They might say when we first had I did take it around everywhere. And a lot of people, even the staff, were saying oh it’s so lovely, it’s so lovely. They liked it, but they wouldn’t need it. You need, that sort of. How can I put it. Less inhibitions. I suppose when you've got dementia, isn't it? Yeah. You know you don't think oh, this is stupid. Because it's a toy. Yeah. You see it as an actual animal. I mean, some people don't they might throw it across the room. You know, that's probably what's happened to the cat.” (Interview_home2)  “Yeah they found them really comforting. Yeah. Yeah. Especially more demented. Residents.” (Interview_home6) |
|  | Limited interest [17] | “Short attention but enjoyed talking to it” (Calendar_home2)  “Enjoyed the dog company for a while before getting bored” (Calendar_home2)  “[Resident] not really interested” (Calendar_home2)  “[Resident] enjoyed the feel of the dog but got fed up and threw it away” (Calendar_home2)  “[Resident] enjoyed initially and then placed on the floor” (Calendar_home3) |
|  | Think it’s real (14) | “One particular lady that I think like we spoke about this, but she would threaten to call the RSPCA because of a cat trying to let the cat outside except for the people that were less involved.” (Interview_home4)  “It is quite incredible, actually how she obviously, I feel that she obviously feels it's real. And like the other particular resident, my mom obviously thinks it's a real cat. And then yeah, so she finds it very, very, very comforting.” (Interview_home7)  “Residents enjoy it, and if I can send you the video of my mum and how she reacts to the cat, you know, and how gentle she is with it, and actually looking into his eyes, you know, and she's talking to it as if it's a real cat.” (Interview_home7)  “There may be moments where he thinks, oh it’s not actually real, but 90% of the time when interacting, he believes it to be real.” (Interview_home8)  “he would respond really well. She would almost think that they're real and really, really calming effect on her, for that instance, is really, really, really effective.” (Interview_home4) |
|  | Dislike (2)[9] | “There is also a resident that doesn't like them, not to that extent, but doesn't like them. And will ask every now and again, can you point it away from me, I don’t like it” (Interview_home5)  “[Resident] does not like cat and didn’t respond well” (Calendar_home2)  “Carried both cat and dog around and said “I’m going to kill these bloody kids”” (Calendar_home4)  “[Resident] likes to look but not touch the dog, dislikes cat” (Calendar_home2) |
|  | Wide appeal (7) | “Yes it’s been brilliant, brilliant. A lot of them are really really keen on them. Really enjoyed having them, some thinking they were real, some realizing they weren't but enjoyed petting them.” (Interview_home1)  “I'll say that they were received by everybody. Yeah. And I think I mean residents that have dementia, I can't say this, you know, for sure. But I would say that they, they feel that they're obviously the residents with dementia, I'm sure that they feel the more realistic, you know, they see them as a real animal in a way. Where the other residents perhaps don’t. But I would say all in all they suit everybody, I will tell you that all in all, to everybody, everybody enjoy using them.” (Interview_home7)  “We've only ever had 19 residents, I'd say about 15 that had at the time, you know, when we participated with them? Yeah, at some point or another?” (Interview_home1)  “You might get a bit either love it, or disinterested. But nothing really negative like that. Nothing severe or saying anything like that at all?” (Interview_home6) |
|  | Reduced mobility (5)[1] | “ay. Took something from it. Yeah. Definitely. Even people that restricted movement, etc. They take into their room if they are like, bed bound and that sort of this. And again, if it had that extra weight, yeah, it would make perhaps a bit more of a difference. Yeah. But yeah, at the end, I think everyone had pretty much positive responses. If the only people that may have been a bit more negative are those that recognize the fact that it wasn’t real okay. But then they can still appreciate it for what it is, if that makes sense. It might not be used on a frequent basis. Yeah, but still topic of conversation. Yeah. Oh, isn't that clever?” (Interview_home8)  “I think the cat sits a bit more nicely on your lap if you're not mobile. Yeah, the dogs a bit heavier.” (Interview_home1)  “We have one lady, quite poorly. And she's still really obsessed with the dog makes its way up there [to the bedroom]” (Interview_home1)  “[Resident] loves the cat, bed bound, adorable moments” (Calendar_home4) |
|  | Previous pets (3)[1] | “He loves that [dog], you know, probably responds to that more than the cat. And that's probably because he had a dog and he loves his dog and his dog came into the garden, you know, and he sees it. It’s been really helpful to him and calming him.” (Interview_home7)  “If they’ve had dogs, they relate to the dog.” (Interview_home2)  “[Resident] had a dog before she was taken ill, she is a great animal lover, she kept the dog all afternoon and evening” (Calendar_home1) |
|  | Infantilising (4) | “They're not not useful for people that just have a mild dementia because they they're just seen as toys.” (Interview_home4)  “We have a couple of negatives. Again, mild dementia a little bit anxious. But the maybe actually a toy, what you're doing, you're talking to a toy, she would make those comments as well, when people engage with the cat or dog or you would invite her over. She’d say, silly people, they’re sat Talking to a toy? That kind of reaction would be We've had a few times. Yeah.” (Interview_home4)  “Yes, absolutely. Because the people who don't have dementia in the home, go, Urgh. Not just not bothered, they think it’s a silly thing.” (Interview_home5)  “There’s stages that this lady goes through where, like, if you go up to her and say, you know, is it okay, don't be silly, you know, they'll be they'll be at different stages during the day where she treats it differently because of how she feels. Yeah, sometimes she knows is completely a robot, it’s a robot don’t be silly.” (Interview_home5) |
|  | Staff dislike (1) | “we’ve had barriers, challenges, one staff member is freaked out, scared of it. So in the lounge, it is a communal area, and we have the cat sitting to the right of the doorway, and the dog is in front of the doorway. And when I come in in the morning, and one of the staff members has been on, they’re both under my desk, because she has to ask them for them to be removed. ” (Interview_home5) |
| Nurture | Cuddled and fussed [29] | “Cuddled as a real one, calmed her down” (Calendar_home1)  “[Resident] missed her real dog and this helped fill the void, she really enjoyed cuddling and fussing the dog” (Calendar_home1) |
|  | Feeding (8)[5] | “Yeah, we did have a lady that enjoyed feeding it. And she had a puree diet.” (Interview_home8)  “Yeah, both, have a good old chat, try feeding them” (Interview_home8)  “They were always very covered in food because they like to be fed.” (Interview_home4)  “I mean, it's been fed many chocolate biscuits, we wet wipe it regularly” (Interview_home5)  “Someone did try feeding hers one day, when they're feeding, because they do still like to play with it. She hasn’t tried to feed it ever before but this one particular day she decided it needed to be fed and was feeding it whatever she was eating. And we had to have a bit of a clean out but it was fine” (Interview_home1)  “We have got one that adores the dog, and he feeds it.” (Interview_home1)  “Sat with them, sort of smoothing them down. One lady wants to feed it, all the time” (Interview_home6)  “Feeding the dog peaches and cream” (Calendar_home4)  “Obsessed with the dog, trying to feed the dog her food” (Calendar_home4) |
|  | Care for and nurture the pet (8)[5] | “we do have one lady who likes to take it into her room, to care for it, she will put it in her bed and cover it over” (Interview_home7),  “And because it is something that he loves and cared for. Not always, but majority of the time. He will soften, lower his tone, start referring to the cat. Very positive for his behaviour really.” (Interview_home8)  “However, having the robot pets really helped, certainly helped with residents, you know, to keep them calm and focus on on actually having a little animal there to care for and look after and comfort them you know” (Interview_home7)  “She's, she's lovely. And she was very caring about the cat. So it seems like they bring out different things in different people.” (Interview_home2)  “I mean, [resident] loved that cat. It was her cat. She would look after it.” (Interview_home2)  “Well, there was an element of worry for it when it was alive and moving, do we need to do anything we need to take it out, does it the need feeding. I mean, it's been fed many chocolate biscuits, we wet wipe it regularly” (Interview_home5)  “That decline, you know, and then she’d say what's wrong with it. I don't know what's wrong with it just because it wasn't moving, not because it wasn’t there” (Interview_home5)  “[Resident] was obsessed with the dog, trying to feed dog her food, got upset when it wasn’t eating” (Calendar_home4)  “And I'll just say to him, oh, can you just? Can you just keep an eye on the on the dog or the puppy? For me, just for five minutes and he’ll sit and talk to it.” (Interview_home7) |

**Adoption**

The evidence in diaries and calendars strongly supported good robot adoption into the services, and usually by particular ‘adoptee’ residents. Staff noted often that residents “*loved the cat*” (Calendar_home3) or “*loved the dog*” (Calendar_home2), “*as if it was her own*” (Calendar_home4). Staff felt it “*was almost emotional*” (Interview_home4) watching residents respond to robots. The second most common code was *Ownership,* with certain residents believing “*they were actually their real pet, claim to take possession of them*” (Interview_home1). Care staff described the pets as “*completely and utterly adopted*” (Interview_home5), suggesting good acceptability. Residents often decided on *Naming* the pets, such as “*he’s Ben isn’t he and so he shall be forevermore*” (Interview_home2), or *Personalising* them with “*a little pink bow in his hair*” (Interview_home5). Congruent with residents ‘adopting’ pets, evidence suggested mostly *Individual Use* rather than group sessions. This seemed to result from particular suitability of devices to some, while other residents had a “*nonplussed reaction*” (Interview_home5), meaning “*we don’t need to circulate it around. It’s useful for certain people, so it’s no good sort of having it as house pet*” (Interview_home5). The residents who benefited most were those not “*having conversations with other residents, […] with dementia*” (Interview_home1), or “*in their rooms*” (Interview_home6). There were 23 counts of evidence towards *Individual Use*, and only five towards *Group Sessions.* Evidence suggested a *High Level of Usage,* with residents keeping *“the dog all day”* (Calendar_home1), and going “*to bed with it*” (Calendar_home2).

A consequence of ‘ownership’ however, was occasions of *Jealousies or Possessiveness* which “*can be quite challenging*” (Interview_home7), as residents were reluctant “*to give it back*” (Interview_home7). There were 12 counts of jealousies or possessiveness across calendars and interviews, with residents reported to “*dominate*” the pets (Calendar_home2), even attempting to report staff for “*animal cruelty*” if the pet was removed (Calendar_home4). While these reports are undoubtedly negative, they also demonstrate strong attachments formed by residents. There appeared to be *No Novelty* effect, with interviews following eight months suggesting “*you can see the love in her eyes every day, when she stroked it this morning, there’s no change in how much she adores it*” (Interview_home5). Some staff felt this effect may result from dementia, meaning residents do not get “*bored*” (Interview_home8), being “*just as interested in them as they ever were*” (Interview_home7).

**Wellbeing effects, particularly mood**

The interviews and calendars strongly supported wellbeing benefits, with 30 counts of evidence towards the code, *Calming*. Staff suggested pets “*had a calming effect*” (Interview_home8), and “*aided*” residents (Interview_home1). The pets were used “*for de-escalation*” with “*residents that are anxious*” to “*prevent them from getting any worse, it will calm them down and help distract them from having a bit of a meltdown*” (Interview_home7). This was supported through 16 counts of *Anxiety Reduced,* with staff reporting “*we had one particular lady that it worked for every single time, it lowered her anxiety*” (Interview_home4). Additionally, when residents were “*missing own dog*” robot provision meant they were “*less stressed*” (Calendar_home1), with others calming after becoming “*unsettled with another resident*” when encouraged to sit “*with her dog*” (Calendar_home4). Similarly, staff also reported pets as *Relaxing* and provided evidence that *Mood Improved* “*because of Covid […] residents […] are missing out on having their own pets. I feel it’s been a God send really having [the robots], especially to […] de-escalate for certain residents*” (Interview_home7). The pets “*lifted quite a few of their moods*” (Interview_home6). Such benefits may have resulted from pets *Providing a Focus,* “*one of the residents goes to her room and the cat goes with her, and it just […] gives her a focus*” (Interview_home5). Similarly, staff reported pets were a *Distraction,* as “*the ones who have dementia that tend to get some of the mood swings […] she will take it which is more of a distraction*” (Interview_home1), thus *Agitation Reduced,* “*[Resident] was feeling very agitat[ed], sat with the dog in lounge and it really calmed her down*” (Calendar_home4). Pets were *Therapeutic,* providing *Reassurance* as “*a tool for giving comfort*” (Interview_home8), with the *“purring”* and *“heartbeat”* both praised in this regard (Interview_home2). The pets promoted *Smiles and Happiness* which staff described as “*wonderful*” (Interview_home7), creating “*lots of smiles*” (Calendar_home4). Overall, reports support that the pets were engaging, with residents having “*a good old chat*” (Interview_home8). The function for robots to *“[turn] on itself*” or interact to noise was praised, for providing “*another activity you can instantly engage with*” (Interview_home8). Consequently, residents were “*more interactive, not falling asleep […] instead […] interacting with the dog and other people about the dog*” (Interview_home2).

**Effects on Communication**

The evidence strongly supported robot impact on residents’ communication, with; the pets, each other, staff and family, further to improving speech capabilities. Residents appeared to enjoy a “*chat*” (Calendar_home4) with the pets, which also provided “*staff and other residents a reason to talk to them*” (Interview_home4), because they give “*you something to discuss […] which sometimes can be quite difficult for some staff*” (Interview_home8). Collaborators felt care staff were “*interacting more*” as pets “*spark conversation*” (Interview_home7), also promoting “*reminiscing*” (Interview_home4). The pets even seemed to improve relationships between “*two ladies who usually spend their day in conflict with each other,*” the staff member suggested the peaceful response “*surprised an old cynic like me*” (Interview_home3).

Some residents were “*able to talk very well, but it’s completely jumbled and it’s really difficult to make sense” “however, when you put the animal in front of them […] then they come out with several, very very clear sentences, so that’s quite critical”* (Interview_home7). Another member of staff reported on “*one of our ladies who has quite severe aphasia*” who when “*engaging solely with the dog shows no signs of this and communicated clearly with it”* (Interview_home3). The potential for robots to improve speech and communication is a profound outcome. The reminiscence involved in the communication is also important, “*it engaged conversations […] about pets that they used to have,” “it’s not just engagement with the animals, it’s also reminiscing about past events as well, which is quite good and a group activity”* (Interview_home4). Interacting with the pets “*gets them to talk about something that’s joyful […] something that they remember with joy*” (Interview_home2).

**Isolation and Covid**

The evidence showed particular benefits of robot pets as a supporting strategy against loneliness and isolation in response to the Covid-19 pandemic. Staff reported “*I’m really glad we have [the pets] especially at this ridiculous time. Yeah. I couldn’t have thought of a better time for us to have them”* (Interview_home5). The pandemic meant care homes were “*not allowed to have any real animals in*” so “*since we’ve had the Covid situation*” the pets have “*come in really, really useful, really useful thing to have*” (Interview_home7). The benefits the pets brought through “*covid*” were “*offering comfort, that little bit of social interaction […] that relationship […] he’s got his friend*” (Interview_home8). The homes also experienced reduced or ceased family visits, with residents “*not seeing their relatives*” (Interview_home6), meaning staff were “*overjoyed*” (Interview_home5) to have the pets as some small alternative. Staff reported “*who knows what [the residents] would have been like throughout lockdown without them. But I feel 100% that [the pets] have improved the situation, from the point of view of yes company, yes a focus*” (Interview_home5). Staff also discussed using the pets for residents self-isolating in their rooms, “*we had a lady in isolation, […] she’s kind of had [the pet] to herself for the whole week. And that’s been really helpful in her isolation period*” (Interview_home4). There was consideration given to infection control however and “*the risk of germs spreading*” (Interview_home5), meaning staff had to be “*more vigilant about cross contamination*” (Interview_home8).

**Design**

Staff suggesting a few possible design improvements based on their experience. One improvement was removable fur, allowing staff to “*take the skin off and wash it or replace it*” (Interview_home8), to be “*fully removable*” (Interview_home7) for hygiene purposes. Staff also felt the dog’s heartbeat could be more pronounced as “*nobody can actually hear it. When you’re holding it, you can’t feel it.*” Although, the dog was praised as being “*sturdier*” with the cat needing more padding, “*is not squishy enough*” (Interview_home2), the *Weight and Size* of the cat was seen as suitable for practical reasons, being “*not so heavy*” as “*a lot of [residents] are […] quite slim built*” (Interview_home1). The dog was considered disadvantaged, being heavier and “*not so easily sat on someone […] because it’s upright*” (Interview_home1).

Staff reported the behaviours of the cat were “*very good*” at making robots look “*more realistic*” (Interview_home1). However, one member of staff felt the dog was less realistic, looking “*like a soft toy*” (Interview_home2). Staff also raised concerns over the noises, with eight counts of staff discussing turning the *Sound Off,*  describing a resident who “*puts up with it for so long […] and then she gets fed up*” (Interview_home1). The cat was described as “*more favourable […] because it’s a little bit quieter*” (Interview_home4). Noises were “*annoying sometimes*” when they sound “*all of a sudden*” “*if you walk past it”* (Interview_home4). Staff reported residents were “*not always wanting the noise on*” (Interview_home1), suggesting “*it could be irritating*” (Interview_home2).

The design was also critiqued in relation to *Expectations*. The cat was perceived as best matching expectations, because it could sit on “*people’s laps*” which is “*normal cat behaviour,*” whereas you wouldn’t normally have a dog “*on the table […] not a dog*” (Interview_home8). Additionally, the dog design was felt incongruent with being a puppy, as “*a puppy wouldn’t sit still […] whereas an older dog will*” (Interview_home8). Thus the design may be better “*if it didn’t look so much like a puppy […] maybe a small dog, a small older dog*” (Interview_home8). Expectations of dog behaviour also meant some residents put their dog “*on the floor, because that’s where she expects a dog to be”* (Interview_home5).

The robustness of devices was questioned, with seven counts of evidence on *Breakages,* for example one dog “*stopped working*” and was replaced, only for the next dog to also encounter a “*problem in it*” where it made beeping and clicking noises (Interview_home5). The cat in another home experienced “*two broken legs*” (Interview_home2).

The discussion on *Battery Life* produced variable evidence, with some homes feeling they “*weren’t too bad actually*” (Interview_home8) and “*were pretty substantial […] we only ever changed them once*” (Interview_home1), while another home “*had to keep changing them because they were used so much”* (Interview_home4).

A number of design features were praised, including the *Purring as Relaxing, Enjoyable Heartbeat,* and the *Importance of Movement*. The movement of the robots was considered important, as a collaborator noticed a resident “*interacted with [broken robot] much less*” and “*responded much less to it when it stopped moving,” “when it broke, […] she sort of lost interest sort of started to ignore it almost when it didn’t move”* (Interview_home5). The purring was praised alongside movement, for being “*quite soothing”* and “*relaxing in itself*” (Interview_home4), with similar comments made around the heartbeat being “*reassuring*” (Interview_home2).

**Suitability**

The data gave some insight into the most suitable use context for use with residents. Generally, staff felt the robots were most accepted and useful for “*people further along*” the dementia journey (Interview_home8). Some staff felt the memory issues encountered with dementia meant “*each time that they see the pet it’s quite new […] they will never grow tired of them*” (Interview_home7). Other homes with varying levels of dementia felt “*more advanced dementia*” residents respond better (Interview_home4). Another home felt that benefiting from the robots required people to “*see it as an actual animal*,” more likely for those with dementia, “*who don’t see it as a toy*” (Interview_home2). Evidence did suggest some residents believed robots were real, with one threatening to “*call the RSPCA*” as the cat wasn’t allowed outside (Interview_home4). The pets also seemed particularly suitable for residents with *Reduced Mobility* who had “*restricted movement*” or were “*bed bound*” (Interview_home8), producing “*adorable moments*” (Calendar_home4) with residents in their rooms and beds.

The limited appeal among residents with no, or mild dementia may also explain the evidence towards *Limited Interest* such as a resident who “*enjoyed the dog company for a while before getting bored*” (Calendar_home2). Regarding *Dislike,* one resident would ask staff to “*point it away from me, I don’t like it*” (Interview_home5). Some of the dislike may be explained through confusion, with one resident carrying the dog and cat around while saying “*I’m going to kill these bloody kids*” (Calendar_home4).

There were also four staff comments around robots as *Infantilising,* as people “*that just have mild dementia*” may see them “*as toys*” (Interview_home4). Another resident with only mild dementia would comment on “*silly people, […] sat talking to a toy*” (Interview_home4). Others reported a robot pet was “*a silly thing*” (Interview_home5). One member of staff also took a dislike, being “*freaked out, scared of it*” and asking “*for them to be removed*” (Interview_home5).

Overall, when asked, staff did however report *Wide Appeal* of the devices, such as “*about 15*” of 16 residents (Interview_home2) or “*more than 90%”* (Interview_home4) enjoying the pets, which did seem more accepted if residents had *Previous Pets,* “*if they’ve had dogs, they relate to the dog*” (Interview_home2).

**Nurture**

The final theme was Nurture, referring to resident’s manor of interaction with the pets. Staff reported that residents *Cuddled and Fussed* the pets, “*cuddled as a real one*” (Calendar_home1), which demonstrates some level of treating the pet as a living animal. There were also 13 counts of residents *Feeding* the pets, including “*peaches and cream*” (Calendar_home4), “*chocolate biscuits*” (Interview_home5), or “*a puree diet*” (Interview_home8). This again shows residents believed pets were real, and an element of care in trying to feed them. The result however was that pets “*were always very covered in food*” (Interview_home4). There were an additional 13 counts of evidence towards the code *Care for and Nurture* such as “*one lady who likes to take it into her room, to care for it, she will put it in her bed and cover it over*” (Interview_home7). Other staff members describe the pet being something a resident “*loves and cared for*” (Interview_home8), and “*would look after*” (Interview_home2).
